# Global, regional, and national burdens of osteoarthritis from 1990 to 2021: findings from the 2021 global burden of disease study

**DOI:** 10.3389/fmed.2024.1476853

**Published:** 2024-11-14

**Authors:** Han-Zheng Li, Xue-Zhen Liang, Yi-Qing Sun, Hai-Feng Jia, Jia-Cheng Li, Gang Li

**Affiliations:** ^1^College of Traditional Chinese Medicine, Shandong University of Traditional Chinese Medicine, Shandong, China; ^2^Orthopaedic Microsurgery, Affiliated Hospital of Shandong University of Traditional Chinese Medicine, Shandong, China

**Keywords:** osteoarthritis, age-standardized rates, incidence, prevalence, disability-adjusted life years

## Abstract

**Background:**

Osteoarthritis (OA) is a common chronic degenerative joint disease and a major contributor to disability and elevated morbidity rates worldwide. This study assesses the epidemiological trends of OA from 1990 to 2021, analyzing data by sex, affected joint sites, and Socio-Demographic Index (SDI) across global, regional, and national levels.

**Methods:**

Data on OA were obtained from the (Global Burden of Disease Study) 2021. The age-standardized rates (ASR) for OA were computed, and the estimated annual percentage changes (EAPC) in ASR were determined to evaluate trends in incidence and disability-adjusted life years (DALYs) over the past three decades. Pearson’s correlation analysis was conducted to explore the relationship between ASR and the Socio-Demographic Index (SDI). Additionally, Joinpoint regression software and age-period-cohort (APC) analysis were applied for a comprehensive examination of the OA data.

**Results:**

From 1990 to 2021, the global burden of OA has markedly increased. In 2021, there were approximately 466.3 million new OA cases, with an ASR of incidence (ASIR) of around 535 per 100,000 population. The prevalence of OA reached about 606.9 million cases, and DALYs rose to approximately 213 million. The burden of OA is significantly higher in women compared to men, as reflected by higher ASR of incidence, prevalence, and DALYs associated with OA. In 2021, the ASR of incidence was positively associated with the SDI regions. Globally, knee osteoarthritis (KOA) remains the most common form of OA. Among the various risk factors, high body mass index (BMI) emerged as the most critical contributor to OA.

**Conclusion:**

From 1990 to 2021, the global burden of OA has steadily increased, leading to a significant decline in health and overall quality of life. The global prevalence of OA indicates higher incidence rates among women and in countries with a higher SDI. Governments and policy makers globally must prioritize increasing awareness of the risk factors and consequences related to OA, promote early diagnostic and therapeutic services, and implement targeted interventions to mitigate the growing burden of OA.

## 1 Introduction

Osteoarthritis (OA) is a common and persistent degenerative joint disease, typically beginning with early signs such as pain and stiffness in the knees, hips, hands, and other joints (1). As the disease progresses, pathological changes such as cartilage damage, bone spur formation, meniscal degeneration, and mild synovitis gradually worsen, potentially leading to restricted movement and joint deformities in advanced stages (2, 3). This severely impacts daily life and functionality, with many patients ultimately requiring joint replacement surgery. Cohort studies have revealed that knee osteoarthritis significantly contributes to work loss and disability, independent of other factors (4). OA is not only one of the most prevalent chronic diseases but also a major cause of disability and high morbidity globally (5). Currently, over 500 million people worldwide suffer from OA, creating a substantial socioeconomic burden (6, 7). Therefore, urgent action is required to mitigate the global burden of OA. Our in-depth analysis of the GBD 2021 database will enhance understanding of the global burden of OA, assess current prevention strategies, and offer policymakers crucial insights into OA prevention and treatment. This will support the efficient allocation of limited healthcare resources and the development of innovative strategies for managing OA.

Previous studies examining the burden of OA and its associated risk factors have been conducted at various levels using data from GBD 2019 or GBD 2020 (8, 9). However, these studies primarily focus on the assessment of OA in select countries, specific anatomical sites, or individual risk factors. To acquire a thorough insight into the recent global burden of OA, our study uses the GBD 2021 database to analyze the prevalence, incidence, and DALYs of OA over the past 30 years at global, regional, and national levels. Additionally, it examines trends by grouping countries and regions based on the SDI to assess the distribution and changes in the global burden of OA.

## 2 Materials and methods

### 2.1 Data source and collection

The GBD database collects and integrates global health-related data from various sources, assessing 371 diseases and 87 risk factors in 204 countries and regions. The database includes eight data indicators: estimate, measure, metric, cause, location, age, sex, and year range (10). The data are chiefly derived from published reports, systematic reviews, data collected from official websites, unpublished original datasets, and contributions from GBD collaborators. The GBD authors are committed to annual updates to ensure the accuracy and reliability of the data (11).

For OA, the GBD reference case definition is symptomatic OA confirmed by radiographic evidence of Kellgren-Lawrence grades 2–4. Grade 2 indicates a definite osteophyte in the joint, grade 3 indicates multiple osteophytes and joint space narrowing, and grade 4 indicates the same as grade 3 plus bone deformity. Symptomatic OA is defined as reported pain for at least 1 month in the past 12 months (12).

DALY is a crucial metric for evaluating the burden of disease, representing the total years lost due to ill-health, disability, or early death. YLD (years lived with disability) is determined by multiplying the number of individuals affected by the duration of the condition until remission or death, adjusted by the disability weight. In contrast, (years of life lost) YLL is calculated by multiplying the total number of deaths by the remaining life expectancy at the age of death according to standard life tables (13). SDI is a composite measure that reflects a country’s development level, incorporating (1) lag-distributed income *per capita* (2) the average years of schooling among the population aged 15 and older, and (3) the total fertility rate under age 25. Based on SDI values, countries and regions are categorized into five groups: high SDI, high-middle SDI, middle SDI, low-middle SDI, and low SDI (14).

### 2.2 Statistical analyses

To analyze the temporal trends in the ASR for incidence and DALYs, we calculated the EAPC. ASR trends are modeled using a logarithmic transformation, expressed as y = *α* + *β*x + *ε*, where y = ln(ASR), x = the calendar year, and ε = the error term, *β* = the ASR trend. The EAPC is calculated using the formula 100 * (exp[β] - 1), with a corresponding 95% confidence interval (CI) to assess statistical significance. ASR is classified as increasing when both the EAPC and the lower end of the 95% CI are above zero. Conversely, the ASR is considered to be decreasing if both the EAPC and the upper end of the 95% CI fall below zero. Otherwise, ASR is considered stable. To identify factors that may influence EAPC, we assessed the relationship between the 2021 APC and SDI and evaluated the relationship between ASRs. The relationships were quantified using the Pearson correlation coefficient (*ρ*). Trend analysis was conducted using Joinpoint regression software (version 4.9.1.0), which established Joinpoint regression models to analyze global trends, stratifying these trends by age group and sex, and the APC and AAPC values were calculated (*p*-value <0.05 was considered statistically significant).

We conducted all data analyses using R software (version 4.3.3), with *p* < 0.05 considered statistically significant. Additionally, AI-powered tools, including OpenAI ChatGPT (15), were employed to facilitate text translation, enhance clarity, summarize information, and correct grammar.

## 3 Results

### 3.1 Temporal trend of OA burden at global level

**Figure 1:**
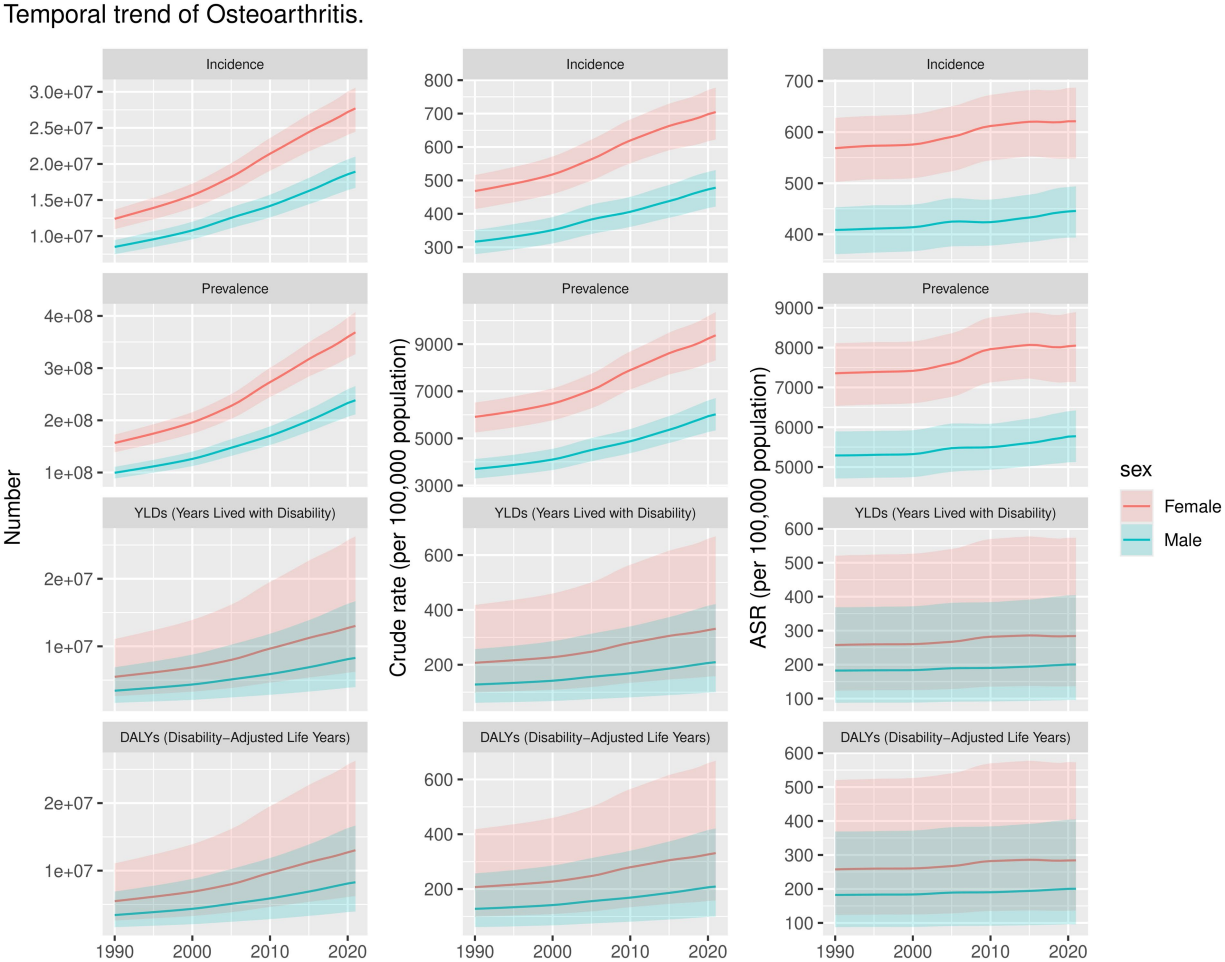
Trends of OA by Incidence, Prevalence, YLDs, DALYs of 27 regions in number, crude rate, and ASR in 1990 and 2021.

The global burden of OA is detailed in Tables 1–4 and Figure 1. In 2021, there were an estimated 466.3 million incident cases of OA worldwide (95% UI: 411.2–516.4 million), corresponding to the ASR of incidence for approximately 535 per 100,000 (95% UI: 472.38–591.97). This ASR has demonstrated a statistically significant increase from 1990 to 2021, with the EAPC of 0.33% (95% CI: 0.31–0.35). The regions with the highest incidence rates were identified in those Middle SDI regions, while Oceania reported the lowest incidence rates in 2021. Regarding prevalence, approximately 606.9 million cases of OA were recorded in 2021 (95% UI: 537.8 to 670.5 million), yielding an ASR of prevalence for 6,967.29 per 100,000 (95% UI: 6,180.7–7,686.06). This prevalence rate has also shown a significant upward trend from 1990 to 2021, with an EAPC of 0.34% (95% CI: 0.31–0.37). Additionally, the DALYs cases associated with OA increased to approximately 213 million (95% UI: 101.8–429.3 million) from 1990 to 2021, reflecting an EAPC of 0.37% (95% CI: 0.33–0.4) (Tables 1–4).

**Table 1:** Incidence of osteoarthritis in 1990 and 2021 for both sexes and all locations, with EAPC from 1990 and 2021.

Location	Num_1990	ASR_1990	Num_2021	ASR_2021	Num_change	EAPC_CI
Andean Latin America	121,137 (107,214–134,640)	519.51 (461.53–577.69)	363,396 (319,748–402,547)	578.18 (511.33–641.1)	2% (1.93–2.07)	0.35% (0.34–0.37)
Australasia	124,186 (110,781–137,553)	555.78 (493.55–617.22)	269,452 (239,374–301,169)	620.09 (550.76–686.53)	1.17% (1.11–1.24)	0.34% (0.31–0.36)
Caribbean	139,108 (123,461–154,387)	513.03 (455.98–570.5)	296,170 (262,547–330,668)	555.77 (493.24–617.51)	1.13% (1.08–1.17)	0.29% (0.27–0.3)
Central Asia	221,869 (194,411–249,130)	444.99 (392.55–498.51)	475,854 (413,121–535,995)	504.46 (442.21–565.75)	1.14% (1.1–1.19)	0.43% (0.4–0.46)
Central Europe	699,280 (618,328–779,466)	474.27 (419.37–525.88)	960,144 (849,389–1,070,329)	522.05 (460.81–580.35)	0.37% (0.35–0.4)	0.33% (0.32–0.35)
Central Latin America	509,648 (450,946–568,026)	527.96 (468.13–587.05)	1,561,605 (1,379,181–1,731,311)	589.49 (521.45–652.52)	2.06% (2.01–2.12)	0.37% (0.36–0.37)
Central Sub-Saharan Africa	122,007 (106,783–135,467)	441.86 (390.35–491.24)	339,701 (298,431–378,043)	463.08 (409.68–515.24)	1.78% (1.72–1.85)	0.12% (0.08–0.16)
East Asia	4,836,774 (4,234,495–5,416,117)	487.3 (428.32–544.04)	12,051,700 (10,562,111–13,556,244)	554.47 (486.91–619.37)	1.49% (1.42–1.56)	0.58% (0.5–0.65)
Eastern Europe	1,495,247 (1,312,342–1,682,319)	550.43 (484.03–614.18)	1,833,038 (1,612,584–2,058,220)	584.97 (515.25–651.42)	0.23% (0.21–0.25)	0.29% (0.26–0.31)
Eastern Sub-Saharan Africa	366,643 (322,665–409,382)	411.5 (363.2–457.47)	1,000,971 (885,084–1,115,451)	461.02 (407.42–509.93)	1.73% (1.68–1.78)	0.39% (0.37–0.4)
Global	20,900,510 (18,467,653–23,104,316)	489.78 (433.1–541.51)	46,632,144 (41,122,053–51,644,431)	535 (472.38–591.97)	1.23% (1.21–1.25)	0.33% (0.31–0.35)
High-income Asia Pacific	1,345,776 (1,189,858–1,491,373)	641.16 (568.18–707.78)	2,189,403 (1,958,142–2,413,152)	682.07 (606.06–752.84)	0.63% (0.58–0.68)	0.35% (0.22–0.49)
High-income North America	1,899,645 (1,695,714–2,093,720)	605.72 (535.69–670.62)	3,457,087 (3,062,826–3,850,766)	646.38 (572.29–715.37)	0.82% (0.78–0.86)	0.07% (−0.07–0.2)
High-middle SDI	5,133,785 (4,537,918–5,675,083)	498.45 (441.11–549.27)	10,350,051 (9,099,042–11,468,242)	548.07 (481.66–608.49)	1.02% (0.99–1.04)	0.38% (0.35–0.42)
High SDI	5,864,566 (5,225,613–6,479,489)	568.82 (505.9–627.74)	10,189,236 (9,088,793–11,269,285)	611.3 (542.71–675.91)	0.74% (0.71–0.76)	0.21% (0.17–0.24)
Low-middle SDI	3,035,073 (2,675,263–3,375,779)	423.51 (374.68–469.5)	7,812,258 (6,892,043–8,676,931)	480.13 (424.96–533.01)	1.57% (1.54–1.6)	0.42% (0.4–0.44)
Low SDI	1,094,622 (965,733–1,219,371)	408.88 (362.19–452.55)	2,804,976 (2,479,498–3,122,532)	447.12 (395.36–493.37)	1.56% (1.53–1.6)	0.29% (0.28–0.31)
Middle SDI	5,750,922 (5,058,232–6,410,278)	475.42 (419.58–527.87)	15,437,969 (13,569,373–17,143,283)	536.49 (473.16–595.02)	1.68% (1.62–1.74)	0.48% (0.44–0.51)
North Africa and Middle East	839,234 (738,099–934,460)	424.31 (375.36–470.67)	2,730,761 (2,402,789–3,044,967)	488.31 (433.7–542.33)	2.25% (2.2–2.3)	0.44% (0.42–0.47)
Oceania	16,659 (14,676–18,607)	441.66 (389.8–491.69)	47,743 (41,835–53,394)	480.95 (423.12–536.36)	1.87% (1.79–1.94)	0.25% (0.22–0.27)
South Asia	2,993,308 (2,640,026–3,326,476)	430.77 (382.03–477.59)	8,220,378 (7,241,991–9,115,916)	495.01 (436.64–548.03)	1.75% (1.7–1.79)	0.47% (0.45–0.49)
Southeast Asia	1,128,950 (993,293–1,259,580)	376.04 (332.26–418.37)	3,261,525 (2,872,405–3,641,133)	437.13 (386.13–485.01)	1.89% (1.83–1.95)	0.51% (0.5–0.51)
Southern Latin America	252,630 (224,153–281,234)	540.18 (478.83–600.89)	483,946 (431,133–536,648)	596.27 (530.39–660.42)	0.92% (0.87–0.96)	0.29% (0.26–0.32)
Southern Sub-Saharan Africa	157,577 (139,016–175,112)	513.31 (454.83–568.81)	375,228 (330,227–417,302)	557.24 (493.49–618.18)	1.38% (1.35–1.41)	0.28% (0.27–0.29)
Tropical Latin America	551,639 (485,844–614,416)	527.59 (467.44–585.25)	1,568,533 (1,385,103–1,733,206)	589.12 (521.39–650.6)	1.84% (1.8–1.89)	0.38% (0.37–0.39)
Western Europe	2,630,868 (2,349,667–2,928,270)	521.51 (465.66–578.73)	3,918,888 (3,498,382–4,363,204)	557.66 (497.27–618.53)	0.49% (0.47–0.51)	0.19% (0.17–0.21)
Western Sub-Saharan Africa	448,326 (395,308–501,301)	439.61 (387.27–489.07)	1,226,622 (1,078,017–1,368,997)	483.84 (427.08–536.68)	1.74% (1.7–1.77)	0.32% (0.3–0.33)

**Table 2:** Prevalence of osteoarthritis in 1990 and 2021 for both sexes and all locations, with EAPC from 1990 and 2021.

Location	Num_1990	ASR_1990	Num_2021	ASR_2021	Num_change	EAPC_CI
Andean Latin America	1,383,606 (1,228,226–1,528,853)	6602.95 (5861.3–7291.11)	4,431,105 (3,933,346–4,885,804)	7370.44 (6552.1–8123.13)	2.2% (2.14–2.27)	0.35% (0.34–0.37)
Australasia	1,659,800 (1,490,005–1,834,682)	7195.28 (6466.74–7950.86)	3,922,514 (3,537,651–4,335,715)	7917.6 (7098.38–8735.71)	1.36% (1.31–1.42)	0.31% (0.29–0.33)
Caribbean	1,698,190 (1,502,561–1,877,189)	6514.84 (5762.16–7,198)	3,852,892 (3,412,425–4,252,980)	7134.56 (6327.26–7876.59)	1.27% (1.23–1.31)	0.32% (0.31–0.33)
Central Asia	2,890,239 (2,514,610–3,288,610)	6143.82 (5368.48–6965.53)	6,010,310 (5,207,826–6,870,321)	7034.89 (6120.08–8010.15)	1.08% (1.04–1.12)	0.47% (0.42–0.51)
Central Europe	9,379,903 (8,274,711–10,510,034)	6276.97 (5554.86–7001.94)	14,519,446 (12,794,343–16,234,191)	6948.51 (6129.15–7752.72)	0.55% (0.53–0.57)	0.36% (0.34–0.37)
Central Latin America	5,697,761 (5,042,112–6,304,605)	6661.13 (5900.48–7353.18)	19,197,614 (16,954,459–21,143,360)	7499.49 (6635.38–8259.93)	2.37% (2.32–2.42)	0.4% (0.39–0.41)
Central Sub-Saharan Africa	1,314,105 (1,159,131–1,464,371)	5622.81 (4965.8–6260.93)	3,517,438 (3,115,894–3,914,394)	5940.49 (5268.27–6589.54)	1.68% (1.61–1.73)	0.13% (0.08–0.18)
East Asia	55,506,569 (48,473,139–62,109,118)	6157.54 (5425.37–6866.85)	158,285,424 (139,469,183–176,824,968)	7036.1 (6216.29–7835.76)	1.85% (1.78–1.91)	0.6% (0.53–0.68)
Eastern Europe	21,046,221 (18,447,721–23,746,861)	7541.08 (6611.08–8496.07)	27,107,908 (23,769,327–30,423,620)	7906.11 (6954.04–8880.09)	0.29% (0.27–0.31)	0.28% (0.24–0.32)
Eastern Sub-Saharan Africa	3,912,597 (3,470,701–4,378,272)	5113.71 (4544.28–5704.44)	10,409,572 (9,265,846–11,557,319)	5829.96 (5160.63–6476.62)	1.66% (1.61–1.71)	0.44% (0.43–0.46)
Global	256,076,700 (227,119,748–283,438,465)	6393.12 (5683.2–7059.53)	606,989,319 (537,873,608–670,519,617)	6967.29 (6180.7–7686.06)	1.37% (1.35–1.39)	0.34% (0.31–0.37)
High-income Asia Pacific	16,572,952 (14,690,574–18,303,601)	8071.98 (7169.35–8905.87)	34,625,938 (31,148,250–37,978,128)	8608.63 (7674.07–9485.19)	1.09% (1.05–1.14)	0.42% (0.25–0.59)
High-income North America	26,834,459 (24,152,314–29,613,959)	7987.16 (7188.9–8824.97)	51,749,679 (46,318,473–57,318,852)	8421.62 (7534.98–9282.03)	0.93% (0.91–0.95)	0.09% (−0.04–0.22)
High-middle SDI	65,704,499 (57,969,290–72,989,870)	6557.5 (5802.43–7265.76)	140,867,094 (124,370,457–156,193,664)	7120.38 (6297.95–7879.76)	1.14% (1.12–1.17)	0.37% (0.33–0.4)
High SDI	79,661,768 (71,442,384–87,921,587)	7371.33 (6609.68–8130.17)	152,390,476 (136,382,303–168,139,764)	7897.27 (7067.13–8689.88)	0.91% (0.9–0.93)	0.24% (0.2–0.29)
Low-middle SDI	33,532,957 (29,739,518–37,224,728)	5326.73 (4722.18–5924.73)	90,721,550 (80,468,538–100,582,854)	6106.25 (5419.32–6763.2)	1.71% (1.67–1.74)	0.45% (0.43–0.47)
Low SDI	11,819,921 (10,482,916–13,238,337)	5080.55 (4507.6–5674.54)	29,900,896 (26,519,031–33,164,829)	5605.58 (4967.54–6230.6)	1.53% (1.5–1.56)	0.32% (0.31–0.34)
Middle SDI	65,080,910 (57,656,741–72,193,595)	6066.13 (5375.33–6744.29)	192,598,780 (170,454,604–213,634,901)	6903.8 (6,123–7643.11)	1.96% (1.9–2.02)	0.52% (0.48–0.56)
North Africa and Middle East	9,337,845 (8,286,676–10,395,164)	5362.22 (4751.55–5979.22)	30,491,685 (27,064,615–33,709,288)	6265.22 (5572.94–6946.23)	2.27% (2.22–2.31)	0.49% (0.45–0.52)
Oceania	176,696 (156,978–197,593)	5637.24 (5022.89–6261.36)	508,189 (451,273–565,165)	6196.48 (5474.55–6895.02)	1.88% (1.8–1.94)	0.28% (0.25–0.3)
South Asia	32,454,745 (28,765,905–35,895,035)	5407.04 (4798.72–5985.59)	96,531,169 (85,576,494–106,691,001)	6326.13 (5612.39–7009.64)	1.97% (1.92–2.03)	0.53% (0.51–0.56)
Southeast Asia	12,662,656 (11,238,788–14,120,228)	4796.58 (4256.53–5377.23)	39,227,935 (34,567,607–43,609,405)	5675.8 (5001.76–6320.89)	2.1% (2.04–2.16)	0.56% (0.55–0.57)
Southern Latin America	3,248,110 (2,899,780–3,599,930)	7001.84 (6250.94–7759.15)	6,538,638 (5,891,362–7,213,429)	7669.24 (6896.46–8466.3)	1.01% (0.98–1.06)	0.29% (0.26–0.31)
Southern Sub-Saharan Africa	1,801,904 (1,590,783–2,000,266)	6559.8 (5794.82–7289.01)	4,289,179 (3,773,086–4,755,346)	7161.23 (6333.34–7951.33)	1.38% (1.36–1.41)	0.31% (0.3–0.32)
Tropical Latin America	6,184,012 (5,480,738–6,851,449)	6604.05 (5863.57–7307.66)	19,391,655 (17,180,012–21,545,741)	7424.65 (6582.79–8,241)	2.14% (2.1–2.18)	0.4% (0.39–0.4)
Western Europe	37,369,611 (33,648,956–41,316,152)	6736.67 (6071.81–7425.01)	59,567,389 (53,848,954–65,960,013)	7113.44 (6407.11–7867.1)	0.59% (0.58–0.61)	0.17% (0.15–0.19)
Western Sub-Saharan Africa	4,944,718 (4,378,452–5,533,517)	5494.22 (4872.02–6124.65)	12,813,642 (11,400,709–14,172,790)	6075.81 (5385.72–6757.27)	1.59% (1.56–1.62)	0.34% (0.32–0.35)

**Table 3:** YLDs of osteoarthritis in 1990 and 2021 for both sexes and all locations, with EAPC from 1990 and 2021.

Location	Num_1990	ASR_1990	Num_2021	ASR_2021	Num_change	EAPC_CI
Andean Latin America	48,494 (23,257–97,370)	231.91 (111.18–466.16)	156,693 (75,061–316,245)	260.94 (125.19–526.82)	2.23% (2.16–2.3)	0.38% (0.36–0.4)
Australasia	58,807 (28,504–118,543)	254.48 (123.19–513.81)	140,906 (69,472–286,856)	283.38 (139.24–577.97)	1.4% (1.32–1.47)	0.34% (0.31–0.36)
Caribbean	59,541 (28,499–120,118)	228.48 (109.52–461.2)	135,591 (64,908–274,656)	251.06 (120.09–508.25)	1.28% (1.24–1.32)	0.34% (0.32–0.35)
Central Asia	101,371 (48,802–204,167)	215.8 (104.18–434.3)	212,365 (101,660–425,622)	249.29 (119.63–500.56)	1.09% (1.05–1.14)	0.5% (0.46–0.55)
Central Europe	327,001 (157,196–659,825)	218.64 (104.72–441.82)	514,447 (248,117–1,044,284)	245.41 (117.63–496.21)	0.57% (0.55–0.6)	0.41% (0.39–0.43)
Central Latin America	197,933 (94,851–399,110)	231.66 (110.81–468.03)	676,745 (322,649–1,368,410)	264.58 (126.15–535.65)	2.42% (2.37–2.48)	0.45% (0.44–0.46)
Central Sub-Saharan Africa	44,741 (21,342–90,400)	191.36 (91.09–387.34)	120,682 (57,012–241,714)	204.3 (97.78–412.23)	1.7% (1.62–1.77)	0.17% (0.12–0.23)
East Asia	1,904,132 (915,864–3,832,084)	211 (102.08–424.58)	5,518,037 (2,633,494–11,061,497)	245.04 (117.45–492.41)	1.9% (1.82–1.97)	0.66% (0.58–0.75)
Eastern Europe	742,363 (355,456–1,508,177)	265.83 (126.81–540.87)	965,580 (465,099–1,951,745)	280.78 (134.37–567.03)	0.3% (0.28–0.32)	0.32% (0.28–0.37)
Eastern Sub-Saharan Africa	132,919 (63,595–267,611)	173.52 (83.3–351.43)	358,151 (171,684–719,704)	200.97 (96.11–405.27)	1.69% (1.64–1.75)	0.51% (0.49–0.52)
Global	8,918,857 (4,264,151–17,983,776)	222.8 (106.65–450.29)	21,304,566 (10,189,161–42,935,420)	244.5 (117.06–493.11)	1.39% (1.37–1.41)	0.37% (0.33–0.4)
High-income Asia Pacific	597,447 (286,964–1,207,310)	291.1 (139.9–587.96)	1,276,815 (611,789–2,579,654)	314.98 (150.55–636.77)	1.14% (1.09–1.2)	0.51% (0.3–0.72)
High-income North America	964,587 (463,191–1,948,963)	286.25 (137.18–576.08)	1,857,796 (900,403–3,761,748)	300.89 (144.87–606.97)	0.93% (0.91–0.95)	0.07% (−0.08–0.21)
High-middle SDI	2,294,596 (1,091,956–4,621,567)	228.99 (109.2–462.13)	4,962,550 (2,367,024–9,974,959)	250.58 (119.78–503.69)	1.16% (1.14–1.19)	0.4% (0.36–0.44)
High SDI	2,845,199 (1,366,268–5,724,655)	262.74 (126–529.15)	5,486,879 (2,648,510–11,052,365)	283.13 (136.04–570.53)	0.93% (0.91–0.95)	0.27% (0.21–0.33)
Low-middle SDI	1,135,604 (547,159–2,278,708)	180.18 (86.93–364.19)	3,110,555 (1,492,430–6,248,885)	209.35 (100.4–422.62)	1.74% (1.7–1.78)	0.5% (0.48–0.53)
Low SDI	398,377 (192,604–798,822)	170.9 (82.56–345.11)	1,018,404 (489,393–2,037,322)	190.93 (91.63–384.26)	1.56% (1.52–1.59)	0.37% (0.35–0.39)
Middle SDI	2,235,431 (1,076,654–4,486,204)	208.27 (100.47–419.68)	6,708,209 (3,204,062–13,465,746)	240.41 (115.09–483.99)	2% (1.94–2.06)	0.57% (0.53–0.61)
North Africa and Middle East	319,580 (153,014–643,843)	183.41 (87.76–371.84)	1,049,857 (503,111–2,115,549)	215.92 (103.37–437.62)	2.29% (2.23–2.34)	0.51% (0.47–0.55)
Oceania	6,038 (2,899–12,078)	192.33 (92.49–385.92)	17,457 (8,450–34,943)	212.87 (102.99–428.52)	1.89% (1.81–1.97)	0.3% (0.28–0.33)
South Asia	1,094,410 (528,656–2,197,896)	181.92 (87.8–368.21)	3,311,236 (1,583,934–6,656,919)	216.9 (104–438.04)	2.03% (1.97–2.09)	0.6% (0.58–0.63)
Southeast Asia	432,058 (206,865–871,588)	163.3 (78.14–329.58)	1,357,628 (645,150–2,713,214)	196.18 (93.56–393.42)	2.14% (2.08–2.21)	0.61% (0.61–0.62)
Southern Latin America	115,112 (54,896–232,022)	247.99 (118.32–499.99)	233,580 (112,227–468,574)	273.53 (131.22–548.73)	1.03% (0.98–1.08)	0.31% (0.28–0.34)
Southern Sub-Saharan Africa	62,841 (30,077–125,999)	229.15 (110.1–460.99)	148,991 (71,571–298,108)	249.45 (120.36–500.63)	1.37% (1.34–1.41)	0.3% (0.29–0.32)
Tropical Latin America	213,399 (102,015–428,853)	228.15 (109.07–460.14)	678,602 (325,615–1,368,810)	259.93 (124.74–524.63)	2.18% (2.13–2.23)	0.45% (0.44–0.46)
Western Europe	1,327,348 (642,543–2,667,248)	238.56 (115.19–479.88)	2,131,317 (1,038,440–4,287,323)	253.58 (123.06–510.55)	0.61% (0.59–0.63)	0.19% (0.17–0.22)
Western Sub-Saharan Africa	168,735 (81,206–339,506)	187.48 (90.32–378.77)	442,087 (212,439–888,884)	210.09 (101.11–424.67)	1.62% (1.58–1.66)	0.39% (0.37–0.4)

**Table 4:** DALYs of osteoarthritis in 1990 and 2021 for both sexes and all locations, with EAPC from 1990 and 2021.

Location	Num_1990	ASR_1990	Num_2021	ASR_2021	Num_change	EAPC_CI
Andean Latin America	48,494 (23,257–97,370)	231.91 (111.18–466.16)	156,693 (75,061–316,245)	260.94 (125.19–526.82)	2.23% (2.16–2.3)	0.38% (0.36–0.4)
Australasia	58,807 (28,504–118,543)	254.48 (123.19–513.81)	140,906 (69,472–286,856)	283.38 (139.24–577.97)	1.4% (1.32–1.47)	0.34% (0.31–0.36)
Caribbean	59,541 (28,499–120,118)	228.48 (109.52–461.2)	135,591 (64,908–274,656)	251.06 (120.09–508.25)	1.28% (1.24–1.32)	0.34% (0.32–0.35)
Central Asia	101,371 (48,802–204,167)	215.8 (104.18–434.3)	212,365 (101,660–425,622)	249.29 (119.63–500.56)	1.09% (1.05–1.14)	0.5% (0.46–0.55)
Central Europe	327,001 (157,196–659,825)	218.64 (104.72–441.82)	514,447 (248,117–1,044,284)	245.41 (117.63–496.21)	0.57% (0.55–0.6)	0.41% (0.39–0.43)
Central Latin America	197,933 (94,851–399,110)	231.66 (110.81–468.03)	676,745 (322,649–1,368,410)	264.58 (126.15–535.65)	2.42% (2.37–2.48)	0.45% (0.44–0.46)
Central Sub-Saharan Africa	44,741 (21,342–90,400)	191.36 (91.09–387.34)	120,682 (57,012–241,714)	204.3 (97.78–412.23)	1.7% (1.62–1.77)	0.17% (0.12–0.23)
East Asia	1,904,132 (915,864–3,832,084)	211 (102.08–424.58)	5,518,037 (2,633,494–11,061,497)	245.04 (117.45–492.41)	1.9% (1.82–1.97)	0.66% (0.58–0.75)
Eastern Europe	742,363 (355,456–1,508,177)	265.83 (126.81–540.87)	965,580 (465,099–1,951,745)	280.78 (134.37–567.03)	0.3% (0.28–0.32)	0.32% (0.28–0.37)
Eastern Sub-Saharan Africa	132,919 (63,595–267,611)	173.52 (83.3–351.43)	358,151 (171,684–719,704)	200.97 (96.11–405.27)	1.69% (1.64–1.75)	0.51% (0.49–0.52)
Global	8,918,857 (4,264,151–17,983,776)	222.8 (106.65–450.29)	21,304,566 (10,189,161–42,935,420)	244.5 (117.06–493.11)	1.39% (1.37–1.41)	0.37% (0.33–0.4)
High-income Asia Pacific	597,447 (286,964–1,207,310)	291.1 (139.9–587.96)	1,276,815 (611,789–2,579,654)	314.98 (150.55–636.77)	1.14% (1.09–1.2)	0.51% (0.3–0.72)
High-income North America	964,587 (463,191–1,948,963)	286.25 (137.18–576.08)	1,857,796 (900,403–3,761,748)	300.89 (144.87–606.97)	0.93% (0.91–0.95)	0.07% (−0.08–0.21)
High-middle SDI	2,294,596 (1,091,956–4,621,567)	228.99 (109.2–462.13)	4,962,550 (2,367,024–9,974,959)	250.58 (119.78–503.69)	1.16% (1.14–1.19)	0.4% (0.36–0.44)
High SDI	2,845,199 (1,366,268–5,724,655)	262.74 (126–529.15)	5,486,879 (2,648,510–11,052,365)	283.13 (136.04–570.53)	0.93% (0.91–0.95)	0.27% (0.21–0.33)
Low-middle SDI	1,135,604 (547,159–2,278,708)	180.18 (86.93–364.19)	3,110,555 (1,492,430–6,248,885)	209.35 (100.4–422.62)	1.74% (1.7–1.78)	0.5% (0.48–0.53)
Low SDI	398,377 (192,604–798,822)	170.9 (82.56–345.11)	1,018,404 (489,393–2,037,322)	190.93 (91.63–384.26)	1.56% (1.52–1.59)	0.37% (0.35–0.39)
Middle SDI	2,235,431 (1,076,654–4,486,204)	208.27 (100.47–419.68)	6,708,209 (3,204,062–13,465,746)	240.41 (115.09–483.99)	2% (1.94–2.06)	0.57% (0.53–0.61)
North Africa and Middle East	319,580 (153,014–643,843)	183.41 (87.76–371.84)	1,049,857 (503,111–2,115,549)	215.92 (103.37–437.62)	2.29% (2.23–2.34)	0.51% (0.47–0.55)
Oceania	6,038 (2,899–12,078)	192.33 (92.49–385.92)	17,457 (8,450–34,943)	212.87 (102.99–428.52)	1.89% (1.81–1.97)	0.3% (0.28–0.33)
South Asia	1,094,410 (528,656–2,197,896)	181.92 (87.8–368.21)	3,311,236 (1,583,934–6,656,919)	216.9 (104–438.04)	2.03% (1.97–2.09)	0.6% (0.58–0.63)
Southeast Asia	432,058 (206,865–871,588)	163.3 (78.14–329.58)	1,357,628 (645,150–2,713,214)	196.18 (93.56–393.42)	2.14% (2.08–2.21)	0.61% (0.61–0.62)
Southern Latin America	115,112 (54,896–232,022)	247.99 (118.32–499.99)	233,580 (112,227–468,574)	273.53 (131.22–548.73)	1.03% (0.98–1.08)	0.31% (0.28–0.34)
Southern Sub-Saharan Africa	62,841 (30,077–125,999)	229.15 (110.1–460.99)	148,991 (71,571–298,108)	249.45 (120.36–500.63)	1.37% (1.34–1.41)	0.3% (0.29–0.32)
Tropical Latin America	213,399 (102,015–428,853)	228.15 (109.07–460.14)	678,602 (325,615–1,368,810)	259.93 (124.74–524.63)	2.18% (2.13–2.23)	0.45% (0.44–0.46)
Western Europe	1,327,348 (642,543–2,667,248)	238.56 (115.19–479.88)	2,131,317 (1,038,440–4,287,323)	253.58 (123.06–510.55)	0.61% (0.59–0.63)	0.19% (0.17–0.22)
Western Sub-Saharan Africa	168,735 (81,206–339,506)	187.48 (90.32–378.77)	442,087 (212,439–888,884)	210.09 (101.11–424.67)	1.62% (1.58–1.66)	0.39% (0.37–0.4)

### 3.2 Burden of OA at national level

**Figure 2:**
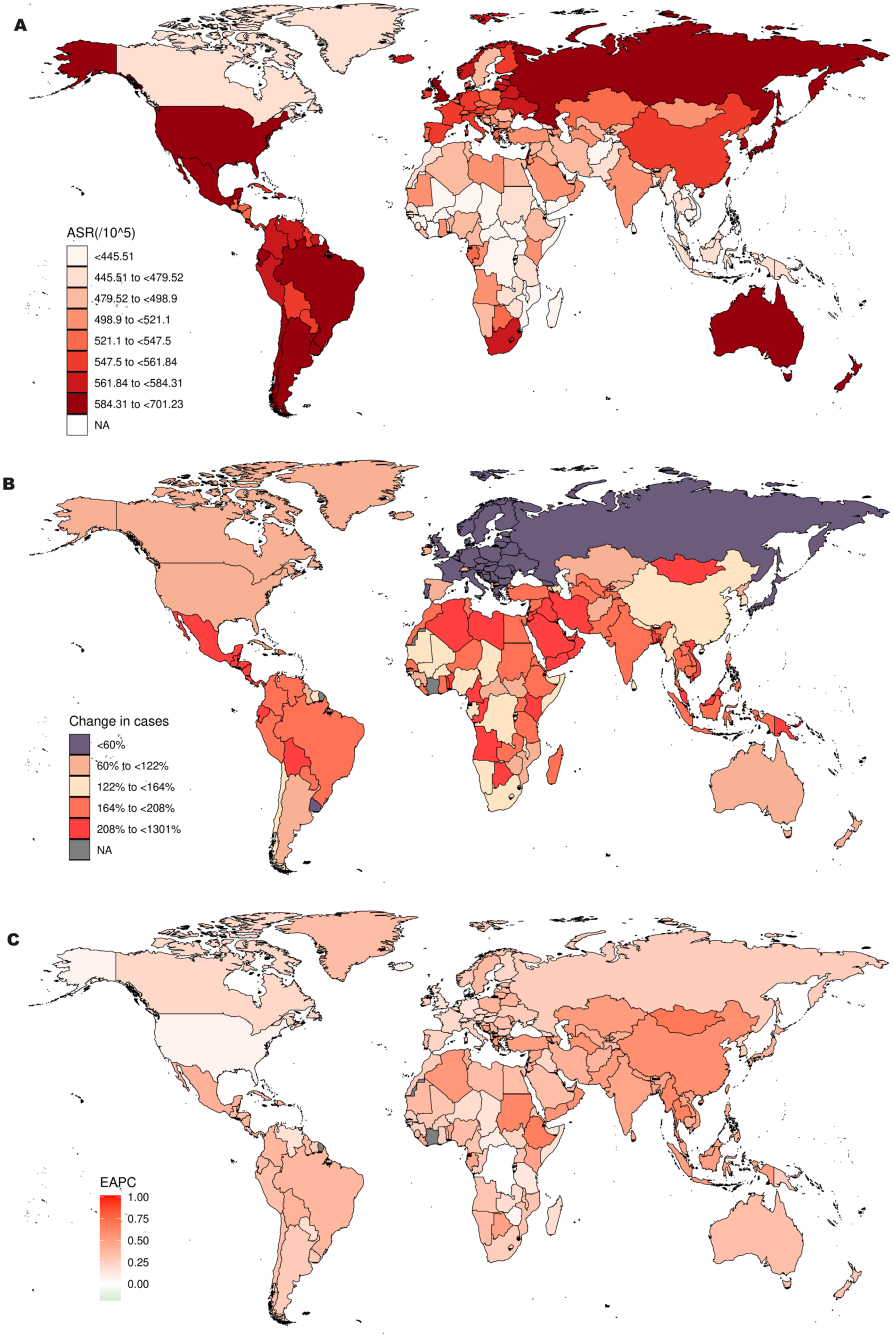
Incidence of OA in 204 countries and regions worldwide burden of disease: (A) ASR of incidence for OA in 2021; (B) Changes in the cases of OA from 1990 to 2021; (C) EAPC analysis of ASR of incidence for OA from 1990 to 2021.

Figure 2 and Supplementary Table S1 summarize the ASR of incidence of OA in 2021, the changes in ASR of incidence at the national level, and the EAPC analysis from 1990 to 2021. First, we examined the ASR of incidence of OA for each country and area in 2021 (Figure 2A). South Korea has the highest ASR at 701.2 per 100,000 (95% UI: 625.36–776.78 per 100,000), followed by Brunei with an ASR of 686.67 per 100,000 (95% UI: 609.4–759.01 per 100,000) and Singapore at 685.67 per 100,000 (95% UI: 606.53–760.52 per 100,000). In contrast, Cambodia has the lowest ASR at 395.3 per 100,000 (95% UI: 350.24–440.74 per 100,000), followed by Timor-Leste with an ASR of 400.38 per 100,000 (95% UI: 352.99–446 per 100,000) and Vietnam at 400.84 per 100,000 (95% UI: 350.75–445.43 per 100,000). Additionally, we analyzed the changes in the number of OA cases in each country and area from 1990 to 2021. As illustrated in Figure 2B, the number of cases in the United Arab Emirates surged by 1,300%, rising from 4,548 in 1990 to 63,707 in 2021. Similarly, Qatar experienced a 973% increase in cases, with numbers growing from 1,287 in 1990 to 13,822 in 2021. However, the number of OA cases in Georgia decreased by 10.6%, from 27,868 in 1990 to 24,890 in 2021 (Figure 2B). Similarly, in Latvia, cases dropped by 2.4%, from 17,577 in 1990 to 17,147 in 2021. These are among the only two countries globally where the incidence rates of OA have declined between 1990 and 2021. To thoroughly evaluate the changes in the ASR of incidence for OA, we conducted an EAPC analysis. The changes in ASR of incidence at the national level are summarized in Figure 2C. Between 1990 and 2021, Equatorial Guinea experienced the highest rate of increase in ASR of incidence, with an EAPC of 1.02% (95% CI: 0.95–1.08), markedly higher than other countries. Mongolia (EAPC: 0.69%; 95% CI: 0.65–0.73) and the Maldives (EAPC: 0.66%; 95% CI: 0.62–0.70) also showed significant increases. Despite the overall upward trend, some countries saw a decrease in ASR of incidence. Israel had a decline with an EAPC of −0.19% (95% CI: −0.36 to −0.03), and the Democratic Republic of the Congo experienced a minimal decrease of −0.003% (95% CI: −0.06 to −0.03). Regionally, East Asia exhibited the fastest rise in ASR of incidence, with an EAPC of 0.58% (95% CI: 0.50–0.65), whereas North America had the slowest growth, with an EAPC of 0.07% (95% CI: −0.07 to 0.20) (Supplementary Table S1).

### 3.3 Percentage trends of incidence changes for different types

**Figure 3:**
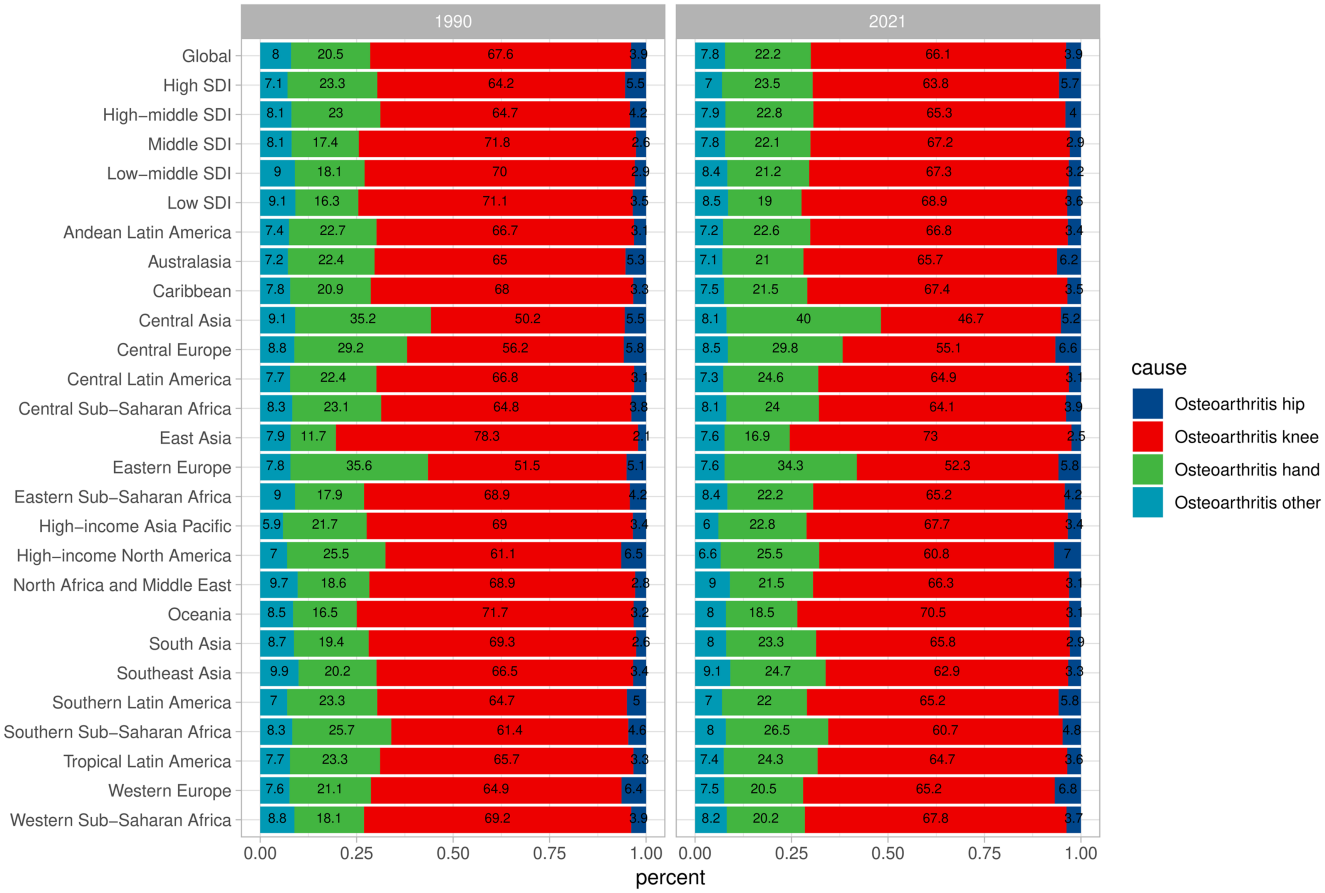
Different types of OA incidence changes globally and regionally in 1990 and 2021.

From 1990 to 2021 the incidence rates of the four types of OA showed little variation, maintaining a relatively stable range of changes. Additionally, on a global scale, knee osteoarthritis is the most common form of the disease, followed by hand osteoarthritis, with hip osteoarthritis and other types of osteoarthritis each accounting for less than 10%. In East Asia, the proportion of knee osteoarthritis cases was 78.3% in 1990 and 73% in 2021. Although there was a decrease, it remains the region with the highest proportion. The proportion of knee osteoarthritis cases in Eastern Europe was 51.5% in 1990 and 52.3% in 2021. Despite the increase, it remains the region with the lowest proportion (Figure 3).

### 3.4 Analysis of factors influencing EAPC

**Figure 4:**
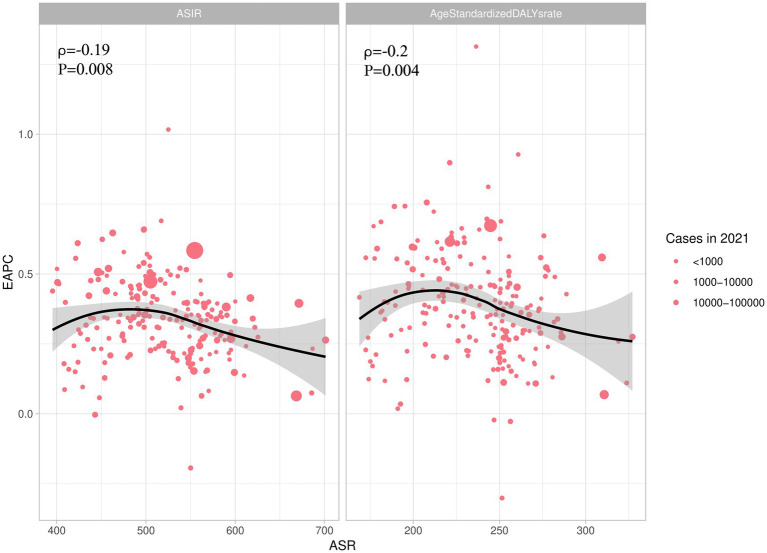
The association between EAPC and ASIR, AgeStandardizedDALYrate. EAPC, Estimated Annual Percent Change; ASIR, ASR of incidence; AgeStandardizedDALYrate, ASR of DALYs.

We conducted an analysis of the factors influencing EAPC, focusing particularly on the ASR of incidence and DALYs. The results revealed an asymmetric inverted V-shaped relationship between EAPC and ASR. Specifically, a negative correlation was observed between the ASR of incidence and EAPC (*ρ* = −0.19; *p* = 0.008) when the ASR exceeded 500 per 100,000. Additionally, when the ASR of DALYs was above 225 per 100,000, there was also a negative correlation between EAPC and ASR (*ρ* = −0.2; *p* = 0.004) (Figure 4).

### 3.5 Trend of OA burden from 1990 to 2021

**Figure 5:**
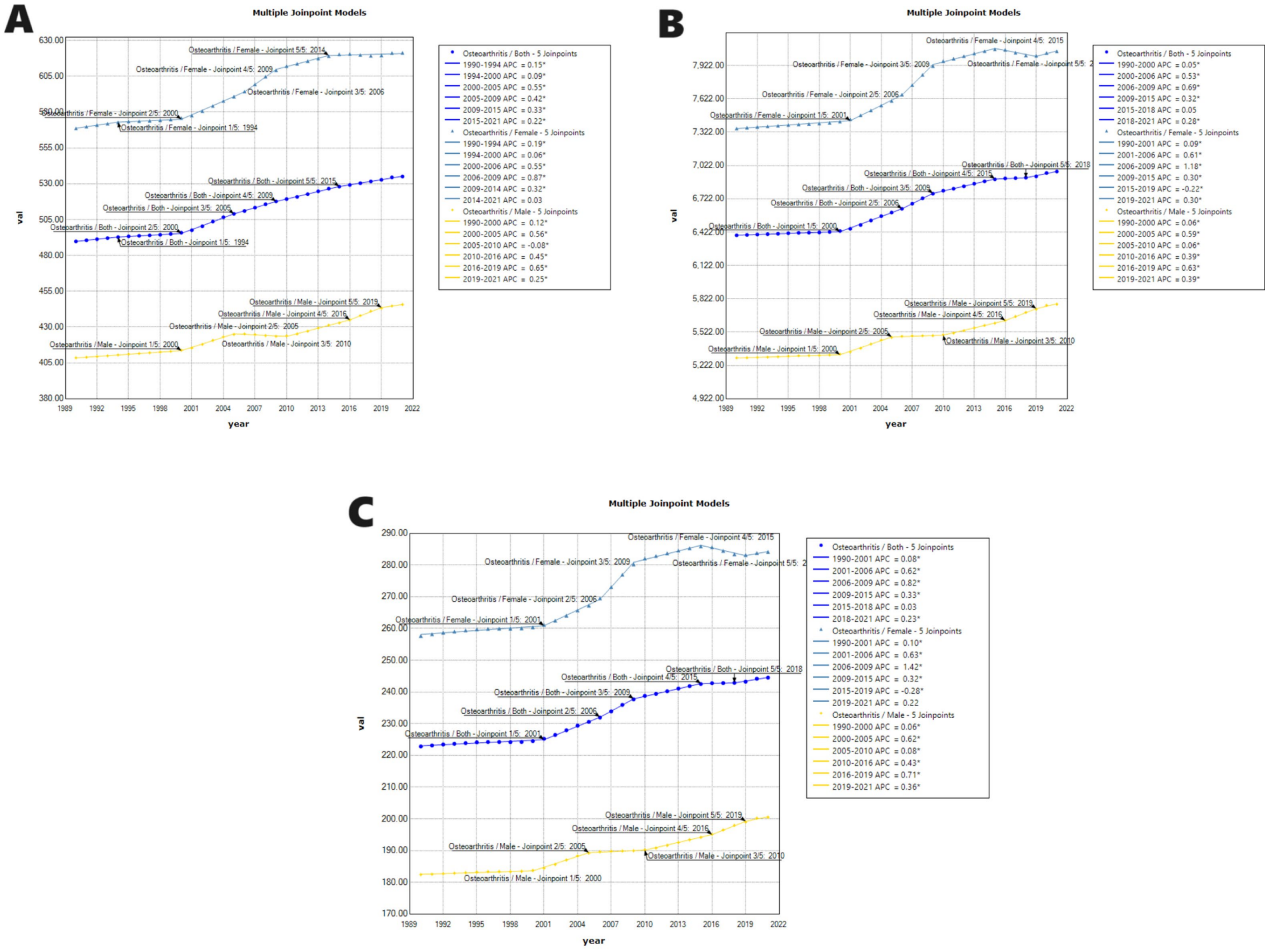
Joinpoint regression analysis of ASR of incidence (A), ASR of prevalence (B), and ASR of DALYs (C) from 1990 to 2021 for OA. APC = annual percentage change.

From 1990 to 2021, the ASR of incidence, ASR of prevalence, and ASR of DALY for OA globally demonstrated a consistent upward trend. The Joinpoint regression model (Figure 5) revealed that the ASR of incidence exhibited a general increase, with an AAPC of 0.8% (95% CI: 0.6–1.1) and a *p*-value <0.001 (Figure 5A). The rate of increase was most pronounced from 2000 to 2005, with an APC of 0.5% (95% CI: 0.5–0.6) and a *p*-value <0.001. Additionally, the ASR of incidence was generally higher in females compared to males. For females, the OA burden showed a decrease from 2010 to 2013, with an APC of −1.4% (95% CI: −2.2 to −0.5) and a *p*-value of 0.004 (Figure 5B). In contrast, the burden for males decreased from 2000 to 2010, with an APC less than 0 and a *p*-value <0.001. The ASR of prevalence increased with an AAPC of 0.3% (95% CI: 0.3–0.3) and a p-value <0.001. Notably, the ASR of prevalence for females decreased from 2015 to 2019, with an APC less than 0 and a *p*-value <0.001. The trend in ASR of DALYs also showed an increase, with an AAPC of 0.3% (95% CI: 0.3–0.3) and a *p*-value <0.001 (Figure 5C). Consistent with ASR of prevalence trends, the burden for females decreased from 2015 to 2019, with an APC less than 0 and a *p*-value <0.001.

### 3.6 The association between ASR and SDI

**Figure 6:**
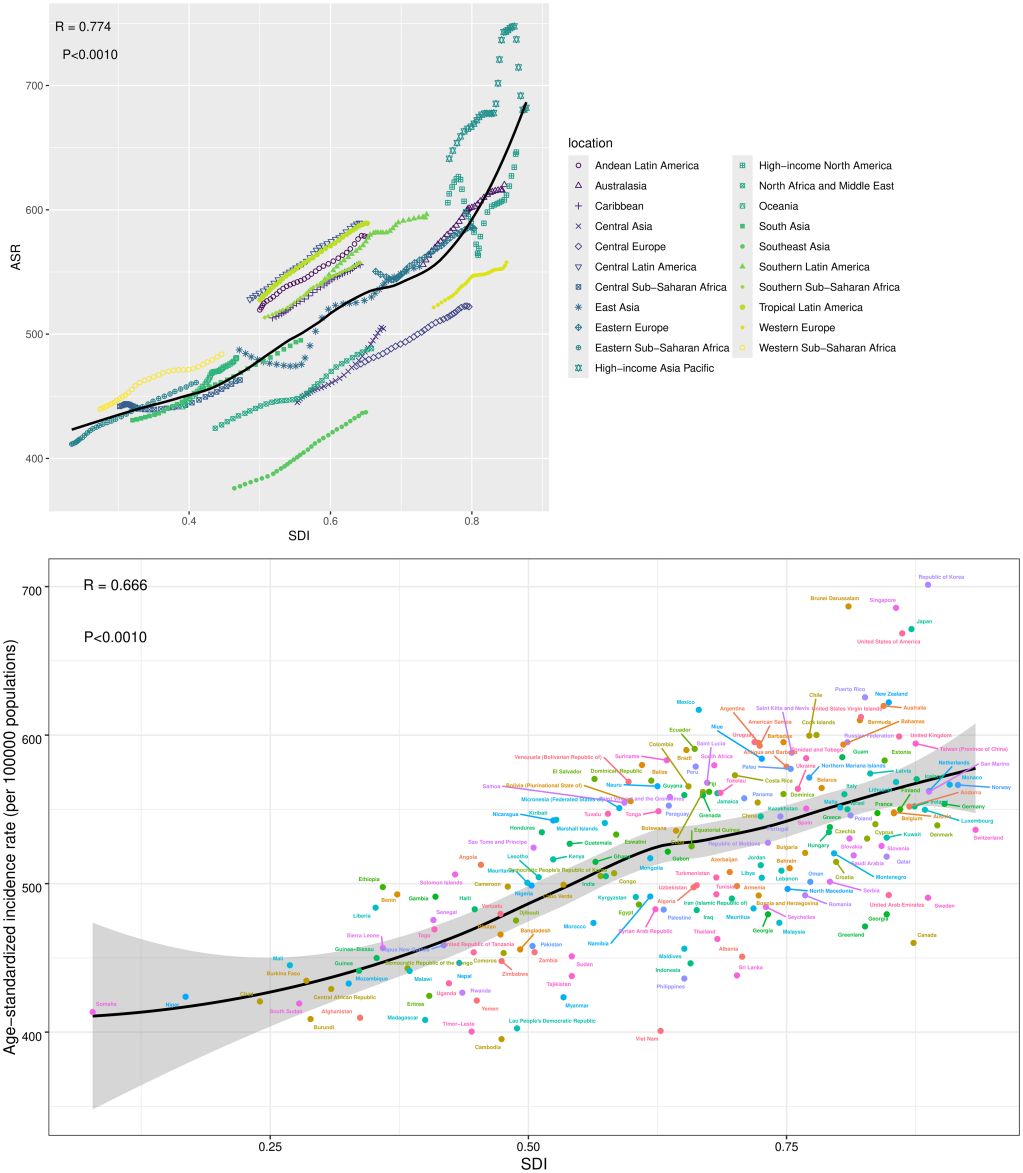
The association between ASR of incidence and SDI at the regions and nations level. SDI, Socio-demographic index.

The findings of the association between ASR of incidence and SDI were detailed in Figure 6, showing a notable positive correlation between the SDI and the ASR of incidence for OA across both regional and national levels from 1990 to 2021 (*R* = 0.774–0.666, *p* < 0.001). This suggests that regions or countries with higher SDI values tend to have higher ASR of incidence for OA. At the regional level, the ASR of incidence levels were higher than expected based on SDI in high-income regions such as Central Latin America, Andean Latin America, Tropical Latin America, Southern Latin America, and Central Asia from 1990 to 2021. At the national level, Japan and the USA also exhibited ASR of incidence levels above those anticipated based on their SDI.

### 3.7 Attributable risk factors for the OA burden

**Figure 7:**
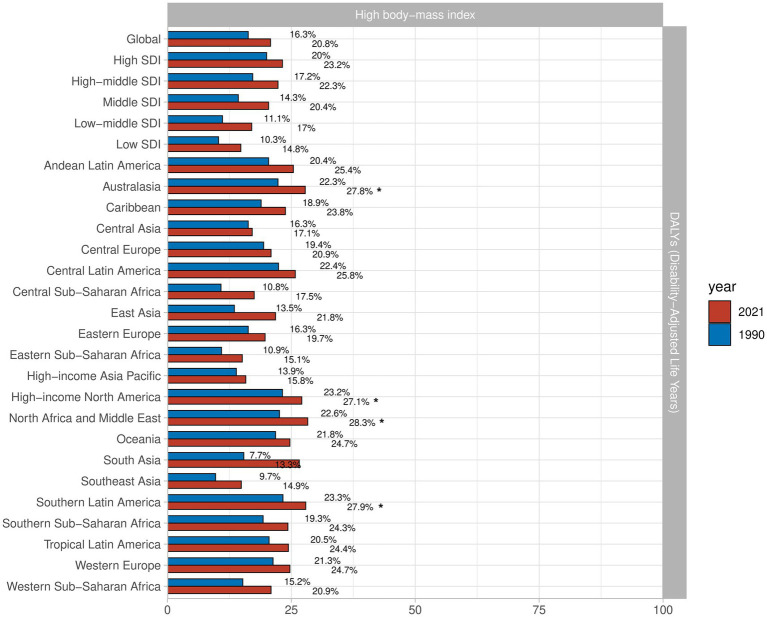
Proportion of DALYs from OA due to high body-mass index in 27 GBD regions in 2021.

To better understand the risk factors for OA, we gathered data on all pertinent risk factors from the GBD 2021. The results, summarized in Figure 7, indicate that high BMI is the primary risk factor for OA. In regions such as North Africa and Middle East, Australasia, High-Income North America, and Southern Latin America, high BMI was identified as the predominant risk factor. Globally, the percentage of DALYs attributed to high BMI has increased significantly by 4.5%. Among regions, East Asia has seen the fastest rise in DALYs attributed to high BMI, with an increase of 8.3%, whereas Central Asia experienced the smallest increase at 0.8%.

## 4 Discussion

OA is ranked as the 18th leading cause of disability globally among the 369 diseases evaluated in the GBD study. The increasing incidence rate and high disability rate of OA impose a significant burden on healthcare systems worldwide (16, 17). While many studies have addressed the global burden of OA using data from the GBD 2019 study (18, 19), we utilize the 2021 GBD data to present the incidence and DALYs associated with OA at the global, regional, and national levels, exploring recent trends and survival patterns over the past 30 years. Besides, we explore the disease burden of OA based on gender, age, and the SDI.

From 1990 to 2021, the ASR of incidence, prevalence, and DALYs for OA has continuously increased. As of 2021, there were approximately 466.3 million new cases of OA, with an ASR of incidence for around 535 per 100,000 population. The prevalence of OA was about 606.9 million cases, and DALYs rose to approximately 213 million. As for the national level, South Korea had the highest ASR of incidence, followed by Brunei and Singapore. Equatorial Guinea experienced the fastest ASR of incidence increase from 1990 to 2021, followed by Mongolia and the Maldives. The study data suggest that the ASR of incidence for OA is associated with the SDI and geographical location of different countries, likely attributable to disparities in socioeconomic conditions, regional factors, and availability of healthcare services (20).

Our research identified KOA as the most common type of OA worldwide, consistent with other studies (21). A study examining knee osteoarthritis epidemiology in the U.S. found that its prevalence increases with each decade of life, peaking in individuals aged 55–64, who experience the highest annual incidence rates (22). An Indian study reported increasing KOA prevalence rates across different age groups: 19.2% for those under 50, 30.7% for those aged 50–60, 39.7% for those aged 60–70, and 54.1% for those over 70 (23). Another study revealed that both the prevalence and incidence of knee osteoarthritis significantly rise after age 55. Among individuals over 55, the average prevalence rate is 13.2%, with 9.4% for men and 18.0% for women (24).

We investigated all risk factors associated with OA and identified high BMI as the primary risk factor. The World Health Organization has proposed three main strategies to address obesity: weight loss, weight maintenance, and prevention of weight gain, all aimed at achieving and maintaining a healthy weight (25). Reyes et al. reported that individuals who are overweight or obese have a significantly elevated risk of developing knee osteoarthritis, with their risk being 2–4 times greater than that of individuals with normal weight. Furthermore, their research highlights a strong association between excess weight and an increased likelihood of OA in the knee, hip, and hand joints (26). An NHANES study revealed that individuals with a healthy weight in early and late adulthood have a reduced risk of developing OA, while those with obesity during these periods have the highest risk (27). Large-scale cohort studies starting from 1990 have also shown that obesity and high BMI are associated with an increased risk of OA progression in the hip, knee, and hand joints (28, 29). High BMI has a significant causal relationship with the risk of weight-bearing joint OA, likely due to mechanical and metabolic changes in the joints of obese adults (30). Overweight places undue stress on joints, leading to degenerative changes (31), while weight loss can mitigate medial tibial cartilage loss, leading to improvements in knee symptoms and overall functional outcomes (32).

Our findings also indicate that the burden of OA is higher in women than in men. OA is more prevalent in women, with the incidence rate doubling in men and tripling in women over the past 20 years in the U.S. (22). Jiang et al. found that women are substantially more prone to developing knee osteoarthritis compared to men, with a 35% increase in KOA risk for every 5-unit rise in BMI (33). A study comparing 610 women with OA from 2004 to 2007 with 3,810 women without OA found that those with the condition had significantly higher body weight and BMI (34). Women in physically demanding, traditionally female jobs, like cleaning, sales, and hairdressing, are at a much higher risk for hip osteoarthritis compared to men. Women also tend to experience more severe OA and a higher rate of related health issues (35, 36).

OA is highly prevalent in the population and increases with age, with no effective cure or significant disease-modifying therapies currently available (37). Individuals with OA are more likely to experience severe comorbidities compared to those without OA (38). Therefore, the prevention and management of OA are crucial. A research center in North America initiated a longitudinal observational study targeting adults aged 45–79 to better assess the development and progression of OA, proposing the Osteoarthritis Initiative (OAI) concept. This initiative includes developing a frailty index, examining its relationship with age and sex, validating mortality, and comparing it with a modified frailty phenotype (39). Measures must be proposed to combat the risk and burden of OA, emphasizing the importance of a healthy lifestyle. In this context, physical activity (PA) plays a crucial role. Regular PA is recognized as a key treatment and management strategy for OA patients, with moderate exercise proven to enhance glycosaminoglycan levels in cartilage, particularly among those at high risk for OA (40, 41). Additionally, providing more counseling, healthcare, psychological education or interventions, and financial support to OA patients is crucial (42).

Our study also has some limitations. One issue is the lack of data from low- and middle-income countries, which may be due to the underdevelopment of healthcare systems and lagging basic research in these regions. Additionally, the 2021 GBD database provides limited information on hand OA, lacking mortality and YLL data for this condition. Furthermore, GBD data are sourced from databases with varying levels of quality, which can introduce heterogeneity and bias. Changes in ICD classifications or misclassification errors may impact the accuracy of the data and subsequent calculations (43).

## 5 Conclusion

Our study offers the most comprehensive and up-to-date evaluation of the global burden of OA from 1990 to 2021, the global burden of OA has continued to rise, significantly impacting health and quality of life. The global prevalence of OA indicates higher incidence rates among women and in countries with a higher SDI. It is imperative that governments and policymakers worldwide enhance awareness of OA risk factors and consequences, promote early diagnostic and therapeutic services, and implement targeted interventions to mitigate the growing burden of OA.

## Data Availability

The original contributions presented in the study are included in the article/Supplementary material, further inquiries can be directed to the corresponding author.
