# Supplementary material for: Global, regional, and national burdens of osteoarthritis from 1990 to 2021: findings from the 2021 global burden of disease study
Source: Front Med (Lausanne). 2024 Nov 14;11:1476853. doi: 10.3389/fmed.2024.1476853 (PMC11602326; doi:10.3389/fmed.2024.1476853)
Supplement: Supplementary file 1 [file Table_1.docx]

Table S1. Incidence of osteoarthritis in 1990 and 2021 for both sexes in 204 countries, with EAPC from 1990 and 2021.

| location | Num_1990 | ASR_1990 | Num_2021 | ASR_2021.x | EAPC_CI | ASR_2021.y |
| --- | --- | --- | --- | --- | --- | --- |
| Afghanistan | 27287 (23791 to 30990) | 369.68 (327.35 to 412.51) | 57027 (49382 to 64359) | 409.68 (362.26 to 460.31) | 0.4% (0.36 to 0.43) | 409.68 (362.26 to 460.31) |
| Albania | 9186 (8124 to 10211) | 394.35 (350.82 to 437.54) | 17461 (15374 to 19597) | 450.79 (399.77 to 501.81) | 0.5% (0.48 to 0.53) | 450.79 (399.77 to 501.81) |
| Algeria | 58393 (51616 to 65377) | 423.18 (375.95 to 471.98) | 209507 (183864 to 233743) | 497.51 (436.82 to 552.44) | 0.54% (0.53 to 0.55) | 497.51 (436.82 to 552.44) |
| American Samoa | 160 (140 to 178) | 547.33 (481.28 to 608.2) | 323 (284 to 361) | 592.89 (525.32 to 658.09) | 0.22% (0.16 to 0.27) | 592.89 (525.32 to 658.09) |
| Andorra | 308 (272 to 344) | 511.64 (452.39 to 572.82) | 806 (715 to 900) | 551.87 (489.74 to 614.22) | 0.23% (0.21 to 0.26) | 551.87 (489.74 to 614.22) |
| Angola | 24001 (21033 to 26933) | 467.32 (411.19 to 518.94) | 83357 (72517 to 93086) | 512.67 (451.42 to 570.53) | 0.31% (0.3 to 0.32) | 512.67 (451.42 to 570.53) |
| Antigua and Barbuda | 262 (235 to 290) | 539.12 (479.32 to 598.36) | 663 (581 to 741) | 578.69 (509.83 to 644.12) | 0.22% (0.21 to 0.23) | 578.69 (509.83 to 644.12) |
| Argentina | 175241 (154713 to 195454) | 541.77 (479.37 to 603.57) | 311890 (277215 to 345792) | 594.76 (527.85 to 658.47) | 0.28% (0.25 to 0.3) | 594.76 (527.85 to 658.47) |
| Armenia | 12390 (10851 to 13990) | 417.83 (366.13 to 467.52) | 19890 (17446 to 22468) | 498.36 (433.96 to 561.53) | 0.66% (0.62 to 0.7) | 498.36 (433.96 to 561.53) |
| Australia | 103535 (92499 to 114683) | 554.29 (492.6 to 615.9) | 225080 (199821 to 251382) | 619.68 (549.23 to 687.01) | 0.34% (0.32 to 0.36) | 619.68 (549.23 to 687.01) |
| Austria | 51885 (46358 to 57455) | 512.67 (455.67 to 567.05) | 78649 (70338 to 87916) | 547.99 (489.83 to 610.91) | 0.19% (0.18 to 0.2) | 547.99 (489.83 to 610.91) |
| Azerbaijan | 25059 (21623 to 28311) | 456.98 (400.36 to 514.64) | 61653 (53118 to 70037) | 507.78 (443.37 to 571.04) | 0.42% (0.35 to 0.49) | 507.78 (443.37 to 571.04) |
| Bahrain | 1352 (1195 to 1500) | 474.08 (417.34 to 525.61) | 8147 (7061 to 9159) | 510.4 (450.11 to 570.18) | 0.23% (0.23 to 0.24) | 510.4 (450.11 to 570.18) |
| Bangladesh | 223684 (198212 to 248765) | 402.6 (355.11 to 449.36) | 690027 (612220 to 767881) | 455.67 (405.41 to 506.44) | 0.44% (0.4 to 0.48) | 455.67 (405.41 to 506.44) |
| Barbados | 1360 (1203 to 1509) | 555.94 (490.84 to 620.48) | 2710 (2387 to 3047) | 595.24 (525.11 to 661.83) | 0.23% (0.21 to 0.25) | 595.24 (525.11 to 661.83) |
| Belarus | 63143 (55568 to 70532) | 507.03 (447.17 to 566.78) | 80484 (70518 to 90198) | 564.41 (497.2 to 630.19) | 0.4% (0.38 to 0.41) | 564.41 (497.2 to 630.19) |
| Belgium | 68466 (60992 to 76513) | 512.44 (456.01 to 570.6) | 97579 (86703 to 108987) | 547.4 (487.41 to 610.97) | 0.18% (0.16 to 0.2) | 547.4 (487.41 to 610.97) |
| Belize | 497 (440 to 550) | 509.91 (450.86 to 565.06) | 2061 (1816 to 2294) | 579.85 (515.88 to 644.57) | 0.39% (0.35 to 0.44) | 579.85 (515.88 to 644.57) |
| Benin | 9508 (8400 to 10546) | 427.31 (379.13 to 475.64) | 32569 (28507 to 36258) | 492.73 (436.01 to 545.17) | 0.46% (0.44 to 0.48) | 492.73 (436.01 to 545.17) |
| Bermuda | 384 (339 to 427) | 584.28 (515.14 to 649.11) | 670 (593 to 746) | 610.02 (538.06 to 674.79) | 0.14% (0.12 to 0.15) | 610.02 (538.06 to 674.79) |
| Bhutan | 1259 (1112 to 1409) | 409.45 (363.49 to 454.69) | 3181 (2820 to 3532) | 465.74 (411.24 to 516.82) | 0.43% (0.41 to 0.44) | 465.74 (411.24 to 516.82) |
| Bolivia | 18351 (16218 to 20443) | 491.71 (436.14 to 549.3) | 57030 (50392 to 63359) | 555.53 (491.78 to 616.88) | 0.4% (0.39 to 0.41) | 555.53 (491.78 to 616.88) |
| Bosnia and Herzegovina | 19585 (17166 to 21961) | 425.94 (377.16 to 473.08) | 26327 (23133 to 29466) | 492 (431.49 to 548.58) | 0.53% (0.47 to 0.59) | 492 (431.49 to 548.58) |
| Botswana | 3012 (2675 to 3379) | 456.76 (405.55 to 509.55) | 10298 (9103 to 11420) | 535.62 (473.79 to 595.78) | 0.49% (0.47 to 0.51) | 535.62 (473.79 to 595.78) |
| Brazil | 538758 (474541 to 599988) | 527.73 (467.49 to 585.46) | 1533313 (1353748 to 1694670) | 589.96 (522.15 to 651.51) | 0.38% (0.37 to 0.39) | 589.96 (522.15 to 651.51) |
| Brunei | 907 (797 to 1004) | 638.1 (565.55 to 711.02) | 3204 (2818 to 3555) | 686.67 (609.4 to 759.01) | 0.23% (0.21 to 0.26) | 686.67 (609.4 to 759.01) |
| Bulgaria | 57146 (50342 to 64003) | 482.22 (426.11 to 535.83) | 59410 (52594 to 65848) | 520.68 (461.73 to 577.32) | 0.25% (0.24 to 0.26) | 520.68 (461.73 to 577.32) |
| Burkina Faso | 19593 (17143 to 21971) | 399.7 (353.47 to 447.89) | 49304 (43079 to 54826) | 434.6 (382.77 to 485.58) | 0.27% (0.26 to 0.27) | 434.6 (382.77 to 485.58) |
| Burundi | 10533 (9293 to 11674) | 399.82 (353.2 to 444.94) | 26112 (22940 to 29320) | 408.81 (361.23 to 455.05) | 0.09% (0.07 to 0.1) | 408.81 (361.23 to 455.05) |
| Cambodia | 18793 (16448 to 21070) | 349.23 (307.33 to 389.74) | 56903 (50231 to 63538) | 395.3 (350.24 to 440.74) | 0.44% (0.42 to 0.45) | 395.3 (350.24 to 440.74) |
| Cameroon | 23980 (21001 to 26765) | 448.31 (396.44 to 501.41) | 82772 (73056 to 92140) | 497.96 (442.52 to 554.89) | 0.31% (0.29 to 0.33) | 497.96 (442.52 to 554.89) |
| Canada | 128828 (114244 to 144138) | 416.01 (365.47 to 464.62) | 263976 (232772 to 297165) | 460.07 (405.52 to 513.81) | 0.21% (0.15 to 0.27) | 460.07 (405.52 to 513.81) |
| Cape Verde | 878 (774 to 988) | 431.97 (381.53 to 485.99) | 2533 (2238 to 2845) | 499.28 (442.48 to 558.22) | 0.49% (0.49 to 0.5) | 499.28 (442.48 to 558.22) |
| Central African Republic | 5884 (5145 to 6581) | 414.92 (365.64 to 461.84) | 13042 (11395 to 14583) | 429.06 (378.82 to 478.07) | 0.1% (0.09 to 0.1) | 429.06 (378.82 to 478.07) |
| Chad | 12181 (10745 to 13631) | 399.77 (352.55 to 447.53) | 30987 (27232 to 34602) | 420.66 (371.48 to 469.65) | 0.15% (0.14 to 0.16) | 420.66 (371.48 to 469.65) |
| Chile | 58214 (51807 to 64573) | 535.97 (476.19 to 595.99) | 145188 (129824 to 162035) | 599.63 (536.35 to 665.62) | 0.33% (0.3 to 0.36) | 599.63 (536.35 to 665.62) |
| China | 4654141 (4075192 to 5212886) | 487.11 (428.13 to 543.75) | 11652721 (10207638 to 13107929) | 554.61 (486.85 to 619.54) | 0.58% (0.51 to 0.66) | 554.61 (486.85 to 619.54) |
| Colombia | 104721 (93002 to 117051) | 506.09 (447.92 to 565.04) | 311333 (273697 to 346614) | 565.49 (498 to 628.15) | 0.38% (0.38 to 0.39) | 565.49 (498 to 628.15) |
| Comoros | 956 (838 to 1072) | 410.18 (362.05 to 460.02) | 2636 (2323 to 2942) | 453.26 (401.04 to 504.17) | 0.34% (0.33 to 0.36) | 453.26 (401.04 to 504.17) |
| Congo | 5796 (5101 to 6479) | 465.42 (408.9 to 518.48) | 19069 (16720 to 21209) | 507.02 (449.56 to 563.6) | 0.27% (0.25 to 0.29) | 507.02 (449.56 to 563.6) |
| Cook Islands | 75 (66 to 84) | 524.97 (466.34 to 583.31) | 141 (124 to 158) | 600.06 (530.08 to 669.97) | 0.4% (0.37 to 0.44) | 600.06 (530.08 to 669.97) |
| Costa Rica | 9941 (8816 to 11032) | 515.07 (456.42 to 573.76) | 31517 (27881 to 35173) | 572.96 (507.23 to 639.61) | 0.35% (0.33 to 0.36) | 572.96 (507.23 to 639.61) |
| Cote d'Ivoire | 23224 (20352 to 25968) | 430.14 (380.01 to 479.29) | 73190 (63804 to 81621) | 473.79 (419.28 to 528.18) | 0.29% (0.27 to 0.3) | 473.79 (419.28 to 528.18) |
| Croatia | 29753 (26249 to 33364) | 475.78 (421.03 to 529.37) | 35691 (31552 to 39616) | 514.63 (454.92 to 573.2) | 0.31% (0.29 to 0.32) | 514.63 (454.92 to 573.2) |
| Cuba | 52466 (46435 to 58324) | 511.3 (452.56 to 569.57) | 99435 (87342 to 111906) | 561.7 (497.53 to 626) | 0.33% (0.32 to 0.35) | 561.7 (497.53 to 626) |
| Cyprus | 4014 (3569 to 4449) | 489.24 (435.53 to 543.47) | 10306 (9254 to 11403) | 540.01 (480.84 to 598.39) | 0.31% (0.29 to 0.33) | 540.01 (480.84 to 598.39) |
| Czech Republic | 64114 (56722 to 72019) | 497.57 (437.85 to 559.98) | 91249 (81004 to 101782) | 530.32 (467.7 to 594.32) | 0.2% (0.19 to 0.22) | 530.32 (467.7 to 594.32) |
| Democratic Republic of the Congo | 82694 (72458 to 92054) | 435.39 (383.37 to 484.02) | 213657 (186934 to 237906) | 442.97 (392.55 to 492.31) | 0% (-0.06 to 0.06) | 442.97 (392.55 to 492.31) |
| Denmark | 36837 (32957 to 40595) | 542.83 (481.96 to 601.04) | 48848 (43441 to 54517) | 539.24 (479.51 to 598.5) | 0.02% (-0.01 to 0.05) | 539.24 (479.51 to 598.5) |
| Djibouti | 781 (687 to 876) | 406.3 (358.06 to 454.02) | 4368 (3830 to 4867) | 475.16 (421.53 to 528.18) | 0.58% (0.54 to 0.62) | 475.16 (421.53 to 528.18) |
| Dominica | 285 (254 to 316) | 516.38 (456.62 to 573.82) | 469 (414 to 523) | 560.33 (497.81 to 619.9) | 0.26% (0.24 to 0.28) | 560.33 (497.81 to 619.9) |
| Dominican Republic | 21754 (19212 to 24251) | 513.72 (455.3 to 568.88) | 60247 (53094 to 67341) | 569.34 (503.36 to 635.73) | 0.36% (0.34 to 0.37) | 569.34 (503.36 to 635.73) |
| Ecuador | 32268 (28496 to 35886) | 533.99 (474.8 to 593.78) | 100807 (88995 to 111969) | 590.72 (522.18 to 656.04) | 0.35% (0.34 to 0.37) | 590.72 (522.18 to 656.04) |
| Egypt | 145297 (127232 to 162962) | 426.67 (376.1 to 479.06) | 395500 (346709 to 441377) | 485.92 (428.33 to 542.21) | 0.35% (0.32 to 0.37) | 485.92 (428.33 to 542.21) |
| El Salvador | 16197 (14204 to 17967) | 506.49 (446.95 to 560.38) | 34374 (30447 to 38032) | 570.47 (505.56 to 631.65) | 0.4% (0.38 to 0.43) | 570.47 (505.56 to 631.65) |
| Equatorial Guinea | 929 (813 to 1044) | 404.63 (357.48 to 451.47) | 3776 (3311 to 4195) | 525.1 (463.69 to 583.57) | 1.02% (0.95 to 1.08) | 525.1 (463.69 to 583.57) |
| Eritrea | 6020 (5275 to 6752) | 387.17 (342.83 to 433.15) | 16113 (14179 to 18023) | 424.42 (373.65 to 470.44) | 0.3% (0.29 to 0.31) | 424.42 (373.65 to 470.44) |
| Estonia | 10258 (9077 to 11434) | 526.34 (465.97 to 586.94) | 11715 (10395 to 13130) | 582.92 (509.57 to 651.05) | 0.35% (0.33 to 0.37) | 582.92 (509.57 to 651.05) |
| Ethiopia | 99638 (87385 to 111462) | 416.45 (368.14 to 462.5) | 270742 (237203 to 300718) | 497.67 (438.54 to 554.72) | 0.66% (0.63 to 0.69) | 497.67 (438.54 to 554.72) |
| Federated States of Micronesia | 267 (237 to 296) | 489.52 (430.63 to 543.96) | 500 (435 to 560) | 550.86 (485.32 to 611.78) | 0.35% (0.31 to 0.4) | 550.86 (485.32 to 611.78) |
| Fiji | 2369 (2091 to 2656) | 489.39 (434.75 to 547.82) | 5075 (4459 to 5702) | 561.84 (494.76 to 627.97) | 0.43% (0.4 to 0.46) | 561.84 (494.76 to 627.97) |
| Finland | 33541 (29882 to 37178) | 512.46 (456.36 to 570.22) | 49030 (43898 to 54528) | 549.98 (493.38 to 610.3) | 0.22% (0.2 to 0.23) | 549.98 (493.38 to 610.3) |
| France | 365821 (325843 to 407123) | 511.42 (453.51 to 571.99) | 558962 (496889 to 624475) | 547.52 (485.99 to 604.87) | 0.2% (0.17 to 0.23) | 547.52 (485.99 to 604.87) |
| Gabon | 2703 (2373 to 3027) | 449.43 (394.82 to 501.74) | 6801 (5969 to 7588) | 521.52 (462.1 to 581.15) | 0.47% (0.45 to 0.48) | 521.52 (462.1 to 581.15) |
| Georgia | 27868 (24387 to 31363) | 446.26 (394.53 to 501) | 24890 (21860 to 27992) | 479.34 (420.92 to 539.34) | 0.2% (0.15 to 0.25) | 479.34 (420.92 to 539.34) |
| Germany | 579625 (515358 to 644919) | 519.99 (464.38 to 575.95) | 787712 (698574 to 873750) | 553.67 (490.76 to 616.73) | 0.15% (0.13 to 0.17) | 553.67 (490.76 to 616.73) |
| Ghana | 37545 (32916 to 41932) | 483.22 (426.33 to 536.33) | 110976 (96895 to 123575) | 514.65 (452.38 to 573.11) | 0.24% (0.16 to 0.32) | 514.65 (452.38 to 573.11) |
| Greece | 68732 (61359 to 77471) | 493.03 (440.17 to 550.49) | 92595 (82686 to 102214) | 538.05 (478.8 to 598.85) | 0.52% (0.42 to 0.62) | 538.05 (478.8 to 598.85) |
| Greenland | 192 (167 to 216) | 421.38 (372.78 to 472.42) | 355 (311 to 402) | 471.16 (416.96 to 527.7) | 0.35% (0.34 to 0.37) | 471.16 (416.96 to 527.7) |
| Grenada | 313 (280 to 346) | 507.06 (448.94 to 563.76) | 682 (602 to 764) | 559.45 (496.57 to 623.76) | 0.31% (0.28 to 0.33) | 559.45 (496.57 to 623.76) |
| Guam | 525 (461 to 586) | 526.14 (459.96 to 585.54) | 1157 (1019 to 1289) | 585.14 (516.21 to 653.49) | 0.35% (0.32 to 0.37) | 585.14 (516.21 to 653.49) |
| Guatemala | 19814 (17426 to 21950) | 473.66 (418.41 to 526.38) | 63652 (56005 to 70928) | 526.84 (464.9 to 587.7) | 0.34% (0.33 to 0.35) | 526.84 (464.9 to 587.7) |
| Guinea | 14609 (12925 to 16366) | 407.52 (360.24 to 458.15) | 29814 (26205 to 33040) | 441.35 (391.03 to 490.61) | 0.23% (0.22 to 0.24) | 441.35 (391.03 to 490.61) |
| Guinea-Bissau | 1959 (1719 to 2194) | 415.91 (366.18 to 466.96) | 4439 (3883 to 4937) | 449.91 (396.64 to 504.3) | 0.23% (0.22 to 0.24) | 449.91 (396.64 to 504.3) |
| Guyana | 2240 (1981 to 2486) | 502.74 (444.6 to 559.47) | 4057 (3554 to 4548) | 559.63 (492.67 to 621.86) | 0.35% (0.33 to 0.37) | 559.63 (492.67 to 621.86) |
| Haiti | 16740 (14742 to 18656) | 442.36 (391.72 to 492.75) | 44247 (39083 to 49489) | 482.72 (427.11 to 537.44) | 0.31% (0.3 to 0.32) | 482.72 (427.11 to 537.44) |
| Honduras | 11446 (10046 to 12716) | 483.21 (423.44 to 537) | 39418 (34602 to 43969) | 534.58 (472.02 to 594.8) | 0.33% (0.33 to 0.34) | 534.58 (472.02 to 594.8) |
| Hungary | 68521 (60691 to 76506) | 500.34 (443.39 to 557.91) | 84065 (74012 to 93556) | 534.71 (469.04 to 597.53) | 0.19% (0.17 to 0.22) | 534.71 (469.04 to 597.53) |
| Iceland | 1419 (1270 to 1570) | 549.74 (490.87 to 609.42) | 2754 (2459 to 3050) | 570.31 (509.81 to 631.84) | 0.08% (0.04 to 0.13) | 570.31 (509.81 to 631.84) |
| India | 2474842 (2182571 to 2747485) | 438.57 (389.25 to 485.34) | 6701764 (5911471 to 7428210) | 505 (445.85 to 559.19) | 0.47% (0.45 to 0.5) | 505 (445.85 to 559.19) |
| Indonesia | 457055 (400852 to 509726) | 383.12 (337.78 to 426.13) | 1308806 (1146853 to 1469965) | 446.32 (393.96 to 496.65) | 0.51% (0.49 to 0.53) | 446.32 (393.96 to 496.65) |
| Iran | 132276 (116401 to 147761) | 431.91 (381.86 to 478.32) | 449414 (395816 to 499367) | 489.96 (433.93 to 541.89) | 0.42% (0.37 to 0.47) | 489.96 (433.93 to 541.89) |
| Iraq | 40791 (35892 to 45168) | 448.04 (392.76 to 498.76) | 149558 (130581 to 167726) | 482.17 (424.64 to 537.96) | 0.21% (0.19 to 0.23) | 482.17 (424.64 to 537.96) |
| Ireland | 18953 (16954 to 20966) | 509.37 (451.56 to 566.39) | 38499 (34403 to 42750) | 551.97 (492.92 to 614.47) | 0.23% (0.22 to 0.25) | 551.97 (492.92 to 614.47) |
| Israel | 23382 (20991 to 25917) | 514.1 (457.68 to 573.02) | 58969 (52884 to 65863) | 550.06 (491.2 to 613.26) | -0.19% (-0.36 to -0.03) | 550.06 (491.2 to 613.26) |
| Italy | 415925 (369369 to 465184) | 528.28 (469.83 to 586.66) | 595991 (530002 to 664509) | 560.17 (498.16 to 622.36) | 0.24% (0.19 to 0.3) | 560.17 (498.16 to 622.36) |
| Jamaica | 8488 (7542 to 9432) | 512.63 (452.87 to 570.6) | 17247 (15256 to 19199) | 560.85 (496.04 to 622.78) | 0.31% (0.29 to 0.33) | 560.85 (496.04 to 622.78) |
| Japan | 1083158 (956010 to 1200741) | 633.39 (560.47 to 699.89) | 1510141 (1348295 to 1664133) | 671.4 (594.22 to 740.18) | 0.4% (0.23 to 0.57) | 671.4 (594.22 to 740.18) |
| Jordan | 7852 (6902 to 8789) | 453.18 (403.15 to 504.64) | 51742 (45552 to 57345) | 512.39 (455 to 567.56) | 0.41% (0.38 to 0.44) | 512.39 (455 to 567.56) |
| Kazakhstan | 64185 (56186 to 72298) | 468.31 (412.41 to 523.97) | 109192 (95014 to 122963) | 545.27 (477.21 to 610.87) | 0.52% (0.49 to 0.54) | 545.27 (477.21 to 610.87) |
| Kenya | 43389 (38340 to 48283) | 447.59 (396 to 495.11) | 151394 (132775 to 168327) | 516.33 (457.17 to 572.7) | 0.48% (0.45 to 0.51) | 516.33 (457.17 to 572.7) |
| Kiribati | 224 (196 to 249) | 499.13 (440.05 to 557.54) | 496 (436 to 556) | 542.96 (480.76 to 604.78) | 0.22% (0.17 to 0.26) | 542.96 (480.76 to 604.78) |
| Kuwait | 4803 (4240 to 5357) | 472.49 (421.41 to 526.8) | 27284 (23711 to 30553) | 530.97 (470.38 to 590.98) | 0.43% (0.42 to 0.45) | 530.97 (470.38 to 590.98) |
| Kyrgyzstan | 13634 (11891 to 15304) | 441.31 (385.52 to 493.85) | 28256 (24309 to 32133) | 490.97 (425.08 to 552.09) | 0.41% (0.36 to 0.46) | 490.97 (425.08 to 552.09) |
| Laos | 8442 (7403 to 9418) | 353.39 (312.05 to 393.84) | 23167 (20272 to 25801) | 402.53 (353.19 to 448.15) | 0.46% (0.44 to 0.49) | 402.53 (353.19 to 448.15) |
| Latvia | 17577 (15584 to 19601) | 518.03 (458.86 to 574.95) | 17147 (15282 to 19092) | 574.13 (507.9 to 640.01) | 0.37% (0.35 to 0.4) | 574.13 (507.9 to 640.01) |
| Lebanon | 10502 (9259 to 11792) | 435.82 (386.32 to 485.68) | 29681 (26182 to 32856) | 508.74 (448 to 564.39) | 0.5% (0.47 to 0.53) | 508.74 (448 to 564.39) |
| Lesotho | 4036 (3571 to 4498) | 433.46 (383.82 to 484.21) | 6345 (5633 to 7046) | 504.29 (447.08 to 559.17) | 0.53% (0.51 to 0.55) | 504.29 (447.08 to 559.17) |
| Liberia | 5476 (4876 to 6106) | 432.33 (384.14 to 485.35) | 14830 (13025 to 16592) | 483.81 (427.55 to 539.8) | 0.45% (0.41 to 0.49) | 483.81 (427.55 to 539.8) |
| Libya | 10070 (8832 to 11224) | 453.84 (400.43 to 504.46) | 35916 (31462 to 40045) | 503.98 (443.81 to 558.83) | 0.35% (0.32 to 0.38) | 503.98 (443.81 to 558.83) |
| Lithuania | 22207 (19616 to 24958) | 511.24 (452.64 to 572.02) | 24886 (21924 to 27897) | 568.44 (499.89 to 637.71) | 0.4% (0.37 to 0.42) | 568.44 (499.89 to 637.71) |
| Luxembourg | 2604 (2328 to 2913) | 517.72 (462.11 to 579.82) | 5250 (4679 to 5850) | 549.7 (491.73 to 611.82) | 0.17% (0.15 to 0.18) | 549.7 (491.73 to 611.82) |
| Macedonia | 9175 (8091 to 10209) | 453.81 (402.11 to 505.54) | 16080 (14211 to 18002) | 496.39 (437.42 to 552.48) | 0.33% (0.32 to 0.35) | 496.39 (437.42 to 552.48) |
| Madagascar | 22701 (19851 to 25438) | 387.82 (341.63 to 435.9) | 62380 (54683 to 69430) | 408.2 (360.87 to 454.33) | 0.18% (0.17 to 0.19) | 408.2 (360.87 to 454.33) |
| Malawi | 18372 (16139 to 20422) | 401.64 (356.05 to 448.51) | 41599 (36587 to 46466) | 441.17 (389.27 to 493.5) | 0.34% (0.33 to 0.35) | 441.17 (389.27 to 493.5) |
| Malaysia | 46814 (41144 to 52105) | 411.58 (363.02 to 457.65) | 149066 (130992 to 165937) | 473.66 (417.83 to 527.02) | 0.47% (0.46 to 0.49) | 473.66 (417.83 to 527.02) |
| Maldives | 443 (384 to 497) | 394.84 (347.18 to 439.8) | 2285 (1997 to 2542) | 456.17 (400.23 to 506.73) | 0.49% (0.46 to 0.52) | 456.17 (400.23 to 506.73) |
| Mali | 18970 (16612 to 21294) | 404.78 (356.37 to 453.36) | 48917 (42975 to 54786) | 445.03 (394.43 to 496.85) | 0.31% (0.3 to 0.31) | 445.03 (394.43 to 496.85) |
| Malta | 2205 (1957 to 2451) | 514.89 (455.64 to 572.87) | 3974 (3570 to 4417) | 551.31 (491.66 to 613.98) | 0.18% (0.15 to 0.21) | 551.31 (491.66 to 613.98) |
| Marshall Islands | 97 (85 to 107) | 483.8 (425.4 to 539.62) | 254 (222 to 284) | 540.81 (474.6 to 600.25) | 0.33% (0.31 to 0.36) | 540.81 (474.6 to 600.25) |
| Mauritania | 4903 (4315 to 5483) | 442.99 (390.85 to 495.64) | 12721 (11194 to 14181) | 500.54 (441.28 to 558.33) | 0.37% (0.35 to 0.38) | 500.54 (441.28 to 558.33) |
| Mauritius | 3554 (3137 to 3952) | 426.21 (374.78 to 474.79) | 8641 (7560 to 9666) | 483.28 (426.38 to 540.07) | 0.43% (0.42 to 0.44) | 483.28 (426.38 to 540.07) |
| Mexico | 271007 (239140 to 301174) | 549.16 (485.53 to 609.06) | 845739 (741870 to 936364) | 617.03 (544.47 to 681.41) | 0.41% (0.4 to 0.43) | 617.03 (544.47 to 681.41) |
| Moldova | 21214 (18723 to 23577) | 466.87 (413.24 to 517.77) | 28864 (25566 to 32254) | 527.51 (467.44 to 589.45) | 0.5% (0.43 to 0.56) | 527.51 (467.44 to 589.45) |
| Mongolia | 4849 (4251 to 5445) | 421.97 (371.58 to 472.44) | 15754 (13472 to 18099) | 517.1 (448.9 to 583.24) | 0.69% (0.65 to 0.73) | 517.1 (448.9 to 583.24) |
| Montenegro | 3188 (2803 to 3555) | 490.1 (430.27 to 544.71) | 4653 (4083 to 5215) | 520.39 (455.72 to 581.45) | 0.24% (0.23 to 0.26) | 520.39 (455.72 to 581.45) |
| Morocco | 68064 (60212 to 76074) | 430.06 (380.3 to 478.99) | 180998 (159987 to 202804) | 473.51 (419.47 to 527.28) | 0.28% (0.25 to 0.31) | 473.51 (419.47 to 527.28) |
| Mozambique | 28521 (25087 to 31792) | 393.94 (348 to 439.98) | 62053 (54601 to 69191) | 432.72 (382.52 to 481.34) | 0.32% (0.3 to 0.33) | 432.72 (382.52 to 481.34) |
| Myanmar | 94100 (83053 to 104576) | 358.73 (318 to 397.31) | 233234 (205139 to 260511) | 423.47 (373.22 to 470.01) | 0.61% (0.58 to 0.64) | 423.47 (373.22 to 470.01) |
| Namibia | 3250 (2855 to 3639) | 435.73 (384.48 to 486.9) | 8344 (7334 to 9310) | 491.31 (434.64 to 546.38) | 0.35% (0.33 to 0.38) | 491.31 (434.64 to 546.38) |
| Nepal | 44229 (39303 to 49167) | 387.57 (345.59 to 431.61) | 115211 (102658 to 128002) | 446.59 (398.83 to 496.31) | 0.48% (0.45 to 0.51) | 446.59 (398.83 to 496.31) |
| Netherlands | 97291 (87433 to 107938) | 536.48 (482.43 to 594.24) | 154895 (137845 to 173273) | 562.31 (499.26 to 625.49) | 0.06% (-0.01 to 0.14) | 562.31 (499.26 to 625.49) |
| New Zealand | 20651 (18415 to 22849) | 563.05 (500.62 to 625.21) | 44372 (39438 to 49395) | 622.04 (553.44 to 687.72) | 0.31% (0.29 to 0.33) | 622.04 (553.44 to 687.72) |
| Nicaragua | 8668 (7628 to 9609) | 480.78 (422.73 to 533.68) | 30198 (26577 to 33716) | 542.53 (479.59 to 605.28) | 0.4% (0.37 to 0.42) | 542.53 (479.59 to 605.28) |
| Niger | 14144 (12459 to 15854) | 399.91 (353.17 to 443.53) | 43202 (38133 to 48221) | 423.84 (375.53 to 472.84) | 0.18% (0.18 to 0.19) | 423.84 (375.53 to 472.84) |
| Nigeria | 227291 (200074 to 254306) | 450.16 (398 to 500.71) | 593768 (521287 to 662832) | 498.72 (440.84 to 553.16) | 0.35% (0.31 to 0.4) | 498.72 (440.84 to 553.16) |
| North Korea | 90679 (79003 to 103017) | 472.99 (414.87 to 528.27) | 175530 (153632 to 197148) | 505.1 (444.93 to 562.6) | 0.23% (0.21 to 0.24) | 505.1 (444.93 to 562.6) |
| Northern Mariana Islands | 165 (145 to 185) | 525.9 (465.45 to 584.25) | 362 (314 to 410) | 571.41 (503.61 to 639.83) | 0.21% (0.17 to 0.26) | 571.41 (503.61 to 639.83) |
| Norway | 28574 (25449 to 31542) | 527.78 (468.9 to 586.59) | 45755 (40756 to 51004) | 566.43 (504.41 to 629.34) | 0.28% (0.19 to 0.37) | 566.43 (504.41 to 629.34) |
| Oman | 4164 (3668 to 4636) | 423.69 (377.23 to 472.04) | 18373 (16230 to 20499) | 501.17 (444.48 to 559.73) | 0.57% (0.54 to 0.6) | 501.17 (444.48 to 559.73) |
| Pakistan | 249293 (218772 to 280263) | 394.09 (346.61 to 441.36) | 710195 (618261 to 798896) | 457.98 (402.52 to 509.95) | 0.52% (0.5 to 0.54) | 457.98 (402.52 to 509.95) |
| Palestine | 4112 (3617 to 4599) | 431.54 (383.21 to 482.87) | 15981 (14017 to 17901) | 482.51 (423.88 to 538.85) | 0.33% (0.31 to 0.35) | 482.51 (423.88 to 538.85) |
| Panama | 8020 (7082 to 8912) | 489.36 (432.95 to 543.53) | 24714 (21961 to 27431) | 557.46 (495.29 to 619.08) | 0.4% (0.39 to 0.41) | 557.46 (495.29 to 619.08) |
| Papua New Guinea | 9687 (8501 to 10874) | 418.16 (367.93 to 466.12) | 32236 (28233 to 36150) | 458.49 (402.59 to 512.05) | 0.27% (0.26 to 0.28) | 458.49 (402.59 to 512.05) |
| Paraguay | 12881 (11367 to 14300) | 523.21 (462.64 to 581.96) | 35221 (31206 to 39089) | 552.5 (490.16 to 612.86) | 0.18% (0.14 to 0.22) | 552.5 (490.16 to 612.86) |
| Peru | 70517 (62193 to 78558) | 520.82 (462.16 to 579.79) | 205559 (180552 to 227854) | 578.82 (509.4 to 641.16) | 0.34% (0.32 to 0.36) | 578.82 (509.4 to 641.16) |
| Philippines | 136805 (119815 to 152614) | 379.14 (333.45 to 423.79) | 419469 (366178 to 471536) | 436.09 (382.63 to 485.96) | 0.42% (0.39 to 0.45) | 436.09 (382.63 to 485.96) |
| Poland | 208141 (183528 to 232356) | 485.83 (428.33 to 540.86) | 328302 (290505 to 366346) | 545.91 (480.16 to 607.04) | 0.4% (0.38 to 0.41) | 545.91 (480.16 to 607.04) |
| Portugal | 64091 (57342 to 71476) | 499.38 (446.73 to 554.39) | 99154 (88674 to 109697) | 545.22 (488.28 to 604.98) | 0.35% (0.27 to 0.43) | 545.22 (488.28 to 604.98) |
| Principality of Monaco | 275 (246 to 307) | 536.83 (480.55 to 597.82) | 391 (350 to 437) | 566.68 (505.3 to 629.25) | 0.15% (0.14 to 0.16) | 566.68 (505.3 to 629.25) |
| Puerto Rico | 20664 (18419 to 23019) | 579.84 (514.61 to 646.56) | 32519 (29015 to 35959) | 625.47 (556.85 to 693.46) | 0.27% (0.26 to 0.29) | 625.47 (556.85 to 693.46) |
| Qatar | 1288 (1128 to 1449) | 471.63 (416.68 to 524.49) | 13822 (12031 to 15489) | 518.18 (460.61 to 577.47) | 0.25% (0.23 to 0.28) | 518.18 (460.61 to 577.47) |
| Republic of Nauru | 31 (27 to 34) | 498.59 (439.16 to 555.24) | 42 (37 to 47) | 565.58 (496.77 to 627.82) | 0.38% (0.36 to 0.4) | 565.58 (496.77 to 627.82) |
| Republic of Niue | 11 (9 to 12) | 520.49 (460.94 to 581.93) | 12 (11 to 14) | 584.1 (517.25 to 647.85) | 0.36% (0.33 to 0.4) | 584.1 (517.25 to 647.85) |
| Republic of Palau | 59 (52 to 66) | 523.52 (461.21 to 583.2) | 156 (136 to 176) | 577.27 (510.57 to 644.04) | 0.29% (0.26 to 0.32) | 577.27 (510.57 to 644.04) |
| Republic of San Marino | 160 (142 to 178) | 524.79 (466.35 to 586.08) | 314 (280 to 351) | 561.86 (499.88 to 630.44) | 0.2% (0.18 to 0.21) | 561.86 (499.88 to 630.44) |
| Romania | 125423 (110912 to 140299) | 451.25 (400.32 to 499.77) | 151599 (133962 to 168487) | 492.13 (434.73 to 545.08) | 0.32% (0.31 to 0.34) | 492.13 (434.73 to 545.08) |
| Russian Federation | 1004303 (881010 to 1125128) | 567.41 (498.64 to 635.22) | 1283831 (1130169 to 1446184) | 595.22 (523.11 to 664.62) | 0.27% (0.23 to 0.3) | 595.22 (523.11 to 664.62) |
| Rwanda | 13013 (11507 to 14540) | 393.2 (349.16 to 439.83) | 33948 (29699 to 37791) | 426.56 (375.22 to 474.36) | 0.29% (0.27 to 0.31) | 426.56 (375.22 to 474.36) |
| Saint Kitts and Nevis | 169 (150 to 187) | 542.16 (477.32 to 601.08) | 468 (410 to 523) | 588.16 (521.19 to 651.58) | 0.26% (0.25 to 0.28) | 588.16 (521.19 to 651.58) |
| Saint Lucia | 444 (395 to 492) | 507.97 (448.15 to 566.44) | 1385 (1221 to 1539) | 567.98 (502.87 to 628.95) | 0.33% (0.3 to 0.37) | 567.98 (502.87 to 628.95) |
| Saint Vincent and the Grenadines | 348 (312 to 388) | 504.61 (449.56 to 563.01) | 798 (705 to 893) | 558.35 (492.89 to 624.11) | 0.35% (0.34 to 0.37) | 558.35 (492.89 to 624.11) |
| Samoa | 477 (419 to 533) | 501.83 (442.89 to 558.99) | 906 (796 to 1019) | 554.5 (488.2 to 621.23) | 0.32% (0.3 to 0.33) | 554.5 (488.2 to 621.23) |
| Sao Tome and Principe | 299 (264 to 335) | 449.75 (398.28 to 501.9) | 764 (669 to 851) | 524.21 (462.18 to 582.24) | 0.51% (0.49 to 0.53) | 524.21 (462.18 to 582.24) |
| Saudi Arabia | 35182 (30926 to 39296) | 438.33 (387.05 to 487.74) | 184906 (161454 to 206867) | 519.02 (457.66 to 575.01) | 0.34% (0.26 to 0.43) | 519.02 (457.66 to 575.01) |
| Senegal | 16080 (14163 to 17955) | 433.81 (381.84 to 484.04) | 44161 (38546 to 49139) | 475.47 (416.82 to 528.94) | 0.26% (0.25 to 0.28) | 475.47 (416.82 to 528.94) |
| Serbia | 53619 (47008 to 60118) | 454.1 (402.28 to 504.77) | 68586 (60637 to 76248) | 501.27 (442.69 to 560.42) | 0.37% (0.36 to 0.39) | 501.27 (442.69 to 560.42) |
| Seychelles | 232 (206 to 258) | 425.83 (375.95 to 474.19) | 635 (552 to 713) | 484.27 (424.3 to 538.98) | 0.41% (0.38 to 0.45) | 484.27 (424.3 to 538.98) |
| Sierra Leone | 9247 (8198 to 10287) | 410.11 (361.85 to 459.47) | 21582 (18976 to 23937) | 456.66 (405.02 to 507.81) | 0.34% (0.32 to 0.37) | 456.66 (405.02 to 507.81) |
| Singapore | 18009 (15810 to 20088) | 662.46 (583.53 to 735.49) | 60587 (53460 to 67211) | 685.67 (606.53 to 760.52) | 0.07% (0.06 to 0.09) | 685.67 (606.53 to 760.52) |
| Slovakia | 28455 (25090 to 31774) | 493.73 (434.26 to 553.74) | 44551 (39164 to 49636) | 530.37 (465.34 to 589.97) | 0.22% (0.2 to 0.24) | 530.37 (465.34 to 589.97) |
| Slovenia | 11790 (10384 to 13186) | 491.11 (431.9 to 546.56) | 18195 (16123 to 20342) | 525.36 (463.12 to 586.4) | 0.22% (0.2 to 0.24) | 525.36 (463.12 to 586.4) |
| Solomon Islands | 762 (666 to 855) | 444.64 (392.62 to 495.53) | 2344 (2039 to 2634) | 506.17 (444.44 to 563.7) | 0.4% (0.37 to 0.42) | 506.17 (444.44 to 563.7) |
| Somalia | 13591 (11944 to 15234) | 397.57 (350.12 to 443.01) | 35857 (31435 to 40006) | 413.53 (365.08 to 463.06) | 0.16% (0.15 to 0.17) | 413.53 (365.08 to 463.06) |
| South Africa | 125033 (110150 to 139040) | 538.52 (477 to 598.27) | 306662 (269896 to 340955) | 579.68 (513.41 to 643.73) | 0.26% (0.25 to 0.27) | 579.68 (513.41 to 643.73) |
| South Korea | 243702 (215123 to 270409) | 665.91 (592.27 to 739.15) | 615472 (547205 to 688216) | 701.23 (625.36 to 776.78) | 0.26% (0.16 to 0.37) | 701.23 (625.36 to 776.78) |
| South Sudan | 11184 (9858 to 12479) | 391.68 (345.93 to 437.05) | 22010 (19217 to 24633) | 419.3 (370.66 to 468.51) | 0.24% (0.23 to 0.25) | 419.3 (370.66 to 468.51) |
| Spain | 247727 (219689 to 278142) | 509.74 (452.16 to 570.09) | 420976 (376269 to 470250) | 550.53 (490.54 to 613.91) | 0.23% (0.2 to 0.26) | 550.53 (490.54 to 613.91) |
| Sri Lanka | 48932 (42912 to 54503) | 380.91 (336.17 to 423.87) | 117977 (103435 to 131691) | 438.19 (385.54 to 486.66) | 0.48% (0.46 to 0.49) | 438.19 (385.54 to 486.66) |
| Sudan | 39609 (34928 to 44229) | 374.78 (330.89 to 417.35) | 114478 (100690 to 127671) | 451.05 (400.85 to 502.02) | 0.62% (0.58 to 0.66) | 451.05 (400.85 to 502.02) |
| Suriname | 1532 (1338 to 1706) | 539.08 (474.72 to 599.35) | 3896 (3417 to 4354) | 583.07 (515.65 to 644.18) | 0.28% (0.27 to 0.29) | 583.07 (515.65 to 644.18) |
| Swaziland | 1688 (1488 to 1891) | 468.62 (414.64 to 521.03) | 3821 (3367 to 4244) | 533.06 (470.1 to 591.84) | 0.37% (0.33 to 0.41) | 533.06 (470.1 to 591.84) |
| Sweden | 54140 (47892 to 60495) | 448.03 (393.92 to 504.34) | 77849 (68631 to 87510) | 490.5 (429.31 to 550.45) | 0.38% (0.2 to 0.55) | 490.5 (429.31 to 550.45) |
| Switzerland | 46179 (41057 to 51247) | 512.78 (455.29 to 571.81) | 76109 (67951 to 85016) | 536.25 (478.09 to 595.83) | 0.13% (0.12 to 0.13) | 536.25 (478.09 to 595.83) |
| Syria | 25784 (22779 to 28805) | 421.3 (376.92 to 467.98) | 75626 (65837 to 85155) | 482.73 (425.82 to 541.86) | 0.46% (0.44 to 0.47) | 482.73 (425.82 to 541.86) |
| Taiwan (Province of China) | 91954 (80996 to 102954) | 519.32 (456.78 to 584.18) | 223449 (197854 to 249986) | 594.32 (526.06 to 663.52) | 0.5% (0.47 to 0.52) | 594.32 (526.06 to 663.52) |
| Tajikistan | 11678 (10187 to 13143) | 398.52 (351 to 447.99) | 32556 (27991 to 36814) | 437.73 (382.62 to 489.19) | 0.34% (0.29 to 0.39) | 437.73 (382.62 to 489.19) |
| Tanzania | 53272 (46769 to 59337) | 425.28 (376.47 to 472.64) | 145867 (129292 to 162102) | 453.74 (402.88 to 504.99) | 0.13% (0.09 to 0.16) | 453.74 (402.88 to 504.99) |
| Thailand | 165162 (144619 to 183600) | 383.46 (337.94 to 423.76) | 484228 (425932 to 544645) | 462.73 (408.91 to 515.69) | 0.65% (0.63 to 0.66) | 462.73 (408.91 to 515.69) |
| The Bahamas | 993 (876 to 1107) | 560.02 (495.79 to 620.75) | 2722 (2371 to 3044) | 593.64 (522.42 to 658.79) | 0.19% (0.18 to 0.21) | 593.64 (522.42 to 658.79) |
| The Gambia | 1849 (1624 to 2066) | 426.6 (376.61 to 478.64) | 6003 (5267 to 6646) | 491.19 (432.75 to 544.29) | 0.46% (0.45 to 0.47) | 491.19 (432.75 to 544.29) |
| Timor-Leste | 1405 (1232 to 1580) | 348.39 (306.28 to 389.21) | 3682 (3244 to 4098) | 400.38 (352.99 to 446) | 0.52% (0.49 to 0.55) | 400.38 (352.99 to 446) |
| Togo | 6576 (5783 to 7297) | 421.5 (372.31 to 469.4) | 24075 (21234 to 26922) | 469.2 (415.21 to 524.26) | 0.33% (0.31 to 0.35) | 469.2 (415.21 to 524.26) |
| Tokelau | 6 (5 to 7) | 485.33 (427.99 to 541.92) | 8 (7 to 9) | 561.14 (496.02 to 621.88) | 0.48% (0.45 to 0.51) | 561.14 (496.02 to 621.88) |
| Tonga | 303 (265 to 338) | 493.71 (435.22 to 550.03) | 469 (412 to 524) | 548.75 (483.06 to 609.57) | 0.28% (0.23 to 0.33) | 548.75 (483.06 to 609.57) |
| Trinidad and Tobago | 4880 (4312 to 5458) | 542.83 (477.85 to 606.65) | 10980 (9707 to 12243) | 584.43 (517.34 to 650.64) | 0.28% (0.26 to 0.3) | 584.43 (517.34 to 650.64) |
| Tunisia | 23828 (21006 to 26756) | 429.98 (379.47 to 481.71) | 69721 (61710 to 77560) | 492.79 (438.3 to 545.86) | 0.45% (0.44 to 0.46) | 492.79 (438.3 to 545.86) |
| Turkey | 172035 (151350 to 191270) | 432.28 (383.55 to 478.7) | 498940 (442251 to 557033) | 504.54 (447.64 to 564.47) | 0.51% (0.44 to 0.57) | 504.54 (447.64 to 564.47) |
| Turkmenistan | 9070 (7961 to 10235) | 431.32 (381.15 to 485.9) | 24003 (20721 to 27399) | 504.12 (440.58 to 569.61) | 0.56% (0.52 to 0.6) | 504.12 (440.58 to 569.61) |
| Tuvalu | 36 (32 to 40) | 483.16 (425.55 to 536.43) | 61 (53 to 68) | 547.04 (481.1 to 611.49) | 0.37% (0.34 to 0.41) | 547.04 (481.1 to 611.49) |
| Uganda | 29418 (25828 to 33000) | 393.38 (345.16 to 440.69) | 82029 (72113 to 91566) | 432.88 (383.31 to 481.65) | 0.32% (0.31 to 0.32) | 432.88 (383.31 to 481.65) |
| Ukraine | 356545 (312760 to 401590) | 523.44 (460.03 to 582.69) | 386110 (341241 to 431370) | 563.85 (495.98 to 629.08) | 0.27% (0.25 to 0.29) | 563.85 (495.98 to 629.08) |
| United Arab Emirates | 4549 (3970 to 5115) | 442.64 (390.98 to 493.56) | 63708 (55394 to 72162) | 492.29 (435.28 to 547.19) | 0.36% (0.34 to 0.38) | 492.29 (435.28 to 547.19) |
| United Kingdom | 416553 (371985 to 462864) | 553.89 (494.51 to 614.65) | 610070 (544798 to 679814) | 599.09 (535.27 to 664.8) | 0.15% (0.04 to 0.26) | 599.09 (535.27 to 664.8) |
| United States | 1770582 (1582306 to 1951370) | 626.75 (554.47 to 694.08) | 3192702 (2830532 to 3554075) | 668.49 (591.69 to 739.57) | 0.06% (-0.08 to 0.21) | 668.49 (591.69 to 739.57) |
| Uruguay | 19164 (17022 to 21378) | 540.15 (478.19 to 600.17) | 26842 (23942 to 29966) | 595.28 (530.77 to 661.96) | 0.29% (0.27 to 0.31) | 595.28 (530.77 to 661.96) |
| Uzbekistan | 53137 (46259 to 59857) | 435.64 (382.79 to 491.51) | 159659 (137466 to 180475) | 498.85 (434.94 to 559.33) | 0.43% (0.4 to 0.47) | 498.85 (434.94 to 559.33) |
| Vanuatu | 343 (299 to 381) | 429.49 (376.82 to 474.39) | 1056 (931 to 1190) | 479.58 (425.49 to 537.88) | 0.34% (0.34 to 0.35) | 479.58 (425.49 to 537.88) |
| Venezuela | 59833 (53028 to 66428) | 525.72 (464.16 to 586.56) | 180660 (159653 to 201259) | 568.58 (504.58 to 632) | 0.16% (0.1 to 0.21) | 568.58 (504.58 to 632) |
| Vietnam | 145579 (128053 to 162688) | 351.54 (310.19 to 392.73) | 448882 (391923 to 502050) | 400.84 (350.75 to 445.43) | 0.47% (0.45 to 0.49) | 400.84 (350.75 to 445.43) |
| Virgin Islands, U.S. | 580 (510 to 648) | 569.16 (503.11 to 631.1) | 889 (784 to 995) | 612.16 (539.67 to 680.71) | 0.24% (0.22 to 0.27) | 612.16 (539.67 to 680.71) |
| Yemen | 21538 (18961 to 23990) | 363.38 (320.07 to 407.27) | 77885 (68747 to 86681) | 421.25 (371.11 to 470.28) | 0.56% (0.52 to 0.59) | 421.25 (371.11 to 470.28) |
| Zambia | 14992 (13119 to 16795) | 430.51 (379.49 to 480.06) | 42993 (37783 to 48108) | 453.81 (400.9 to 504.97) | 0.18% (0.14 to 0.23) | 453.81 (400.9 to 504.97) |
| Zimbabwe | 20557 (18221 to 22842) | 429.34 (381.87 to 477.08) | 39758 (34864 to 44366) | 447.86 (394.32 to 502.7) | 0.06% (0.02 to 0.09) | 447.86 (394.32 to 502.7) |
